# Phenotypic characterization and genome analysis of a novel *Salmonella* Typhimurium phage having unique tail fiber genes

**DOI:** 10.1038/s41598-022-09733-5

**Published:** 2022-04-06

**Authors:** Sadia Sattar, Inam Ullah, Sofia Khanum, Marc Bailie, Bushra Shamsi, Ibrar Ahmed, Syed Tahir Abbas Shah, Sundus Javed, Aamir Ghafoor, Amna Pervaiz, Fakiha Sohail, Naseer Ali Shah, Kaleem Imdad, Nazish Bostan, Eric Altermann

**Affiliations:** 1 Molecular Virology Labs, Department of Biosciences, Comsats University Islamabad, Islamabad, Pakistan; 2 AgResearch, Palmerston North, New Zealand; 3 Alpha Genomics Private Limited, Islamabad, 45710 Pakistan; 4 Functional Genomics Lab, Department of Biosciences, Comsats University Islamabad, Islamabad, Pakistan; 5 Microbiology and Immunology Lab, Department of Biosciences, Comsats University Islamabad, Islamabad, Pakistan; 6 University Diagnostic Lab, University of Veterinary and Animal Sciences (UVAS) Lahore, Lahore, Pakistan; 7 Riddet Institute, Massey University, Palmerston North, New Zealand

**Keywords:** Bacteriophages, Microbial genetics, Viral genetics

## Abstract

*Salmonella* *enterica* serovar Typhimurium is a foodborne pathogen causing occasional outbreaks of enteric infections in humans. *Salmonella* has one of the largest pools of temperate phages in its genome that possess evolutionary significance for pathogen. In this study, we characterized a novel temperate phage *Salmonella* phage BIS20 (BIS20) with unique tail fiber genes. It belongs to the subfamily *Peduovirinae* genus *Eganvirus* and infects *Salmonella* Typhimurium strain (SE-BS17; Acc. NO MZ503545) of poultry origin. Phage BIS20 was viable only at biological pH and temperature ranges (pH7 and 37 °C). Despite being temperate BIS20 significantly slowed down the growth of host strain for 24 h as compared to control (P < 0.009). Phage BIS20 features 29,477-base pair (bp) linear DNA genome with 53% GC content and encodes for 37 putative ORFs. These ORFs have mosaic arrangement as indicated by its ORF similarity to various phages and prophages in NCBI. Genome analysis indicates its similarity to *Salmonella enterica* serovar Senftenberg prophage (SEStP) sequence (Nucleotide similarity 87.7%) and *Escherichia* virus 186 (~ 82.4% nucleotide similarity)*.* Capsid genes were conserved however those associated with tail fiber formation and assembly were unique to all members of genus *Eganvirus*. We found strong evidence of recombination hotspot in tail fiber gene. Our study identifies BIS20 as a new species of genus *Eganvirus* temperate phages as its maximum nucleotide similarity is 82.4% with any phage in NCBI. Our findings may contribute to understanding of origin of new temperate phages.

## Introduction

*Salmonella* Typhimurium, a major foodborne pathogen with zoonotic potential is responsible for several outbreaks of salmonellosis in humans resulting in high levels of morbidity and economic losses. Most outbreaks are due to the consumption of contaminated livestock and food products^1,2^. The prevalence of these serovars in the food chain leads to high chances of human infections^3^. It is important to study the factors responsible for host adaptability in different ecological niches. *Salmonella* harbors a wide variety of temperate phages in their genomes. The role of temperate phages is critical for host adaptability during infection and for disease epidemiology. Particularly, phages as mobile genetic elements are found to be a source of genetic differences between avirulent and virulent strains of *E. coli* and other members of *Enterobacteriaceae*^4^*.* Prophages integrated into the host genome can encode genes that are not required for phage production instead, they can be of benefit to the host cell. These genes can enhance the bacterial host’s fitness either by producing toxins or by increasing the fitness of the host during infection^5^*.* Organism tracing during a disease outbreak utilizes phage typing and serovar identification to distinguish isolates of the same serovar^6^. For example, recent publications from the United Kingdom reported the presence of a specific prophage in an invasive strain of *Salmonella* Typhimurium responsible for causing salmonellosis, that was not reported before in any UK-based strain^7–9^. Hence temperate phage integration in host genomes exerts a directional pressure on host evolution and adaptability.

Most dsDNA-tailed temperate phages have mosaic genomes originating from different evolutionary backgrounds with interchangeable modules^10^. This mosaic module arrangement implies that mosaic genomes have originated by horizontal gene transfer of different functional modules from a common ancestor^9,11,12^. Mosaic structure is best studied in lambdoid and T4-like bacteriophages^13,14^. Phage genome regions associated with tail fibers exhibit a high degree of mosaicism and are recombination hot spots^15^. These recombination events may be the result of host/ environmental selection pressure on phages for finding new hosts or to adapt to an already evolved host, however, temperate phages may be subjected to completely different types of pressures as faced by lytic phages. Integrated prophages will undergo the same selection pressure as endured by host chromosome. In fact, even defective prophages can confer beneficial phenotypes to the host^16,17^, conversely these defective prophages can also provide functional genes to integrated temperate phages that result in new phage variants capable of infecting new hosts (host range expansion). There is a constant exchange of genes between integrated prophages and temperate phages by homologous recombination that makes the evolutionary pattern more versatile^18^. In this study we isolated and characterized a novel *Salmonella* temperate phage BIS20 that exhibits mosaic genome arrangement and has sequence similarity to *Salmonella* prophage SEStP and *Escherichia* phage 186. However, BIS20 differs from it in repressor system and tail fiber genes. We found a clear evidence of recombination hotspot in tail fiber gene of BIS20 that may contribute to its evolutionary significance by shedding light into the processes that lead to host range changes among bacteriophages. BIS20 may represent an intermediate link between *Salmonella* prophage SEStP and *Escherichia* phage 186.

## Results

### Bacteriophage isolation and characterization

**Figure 1:**
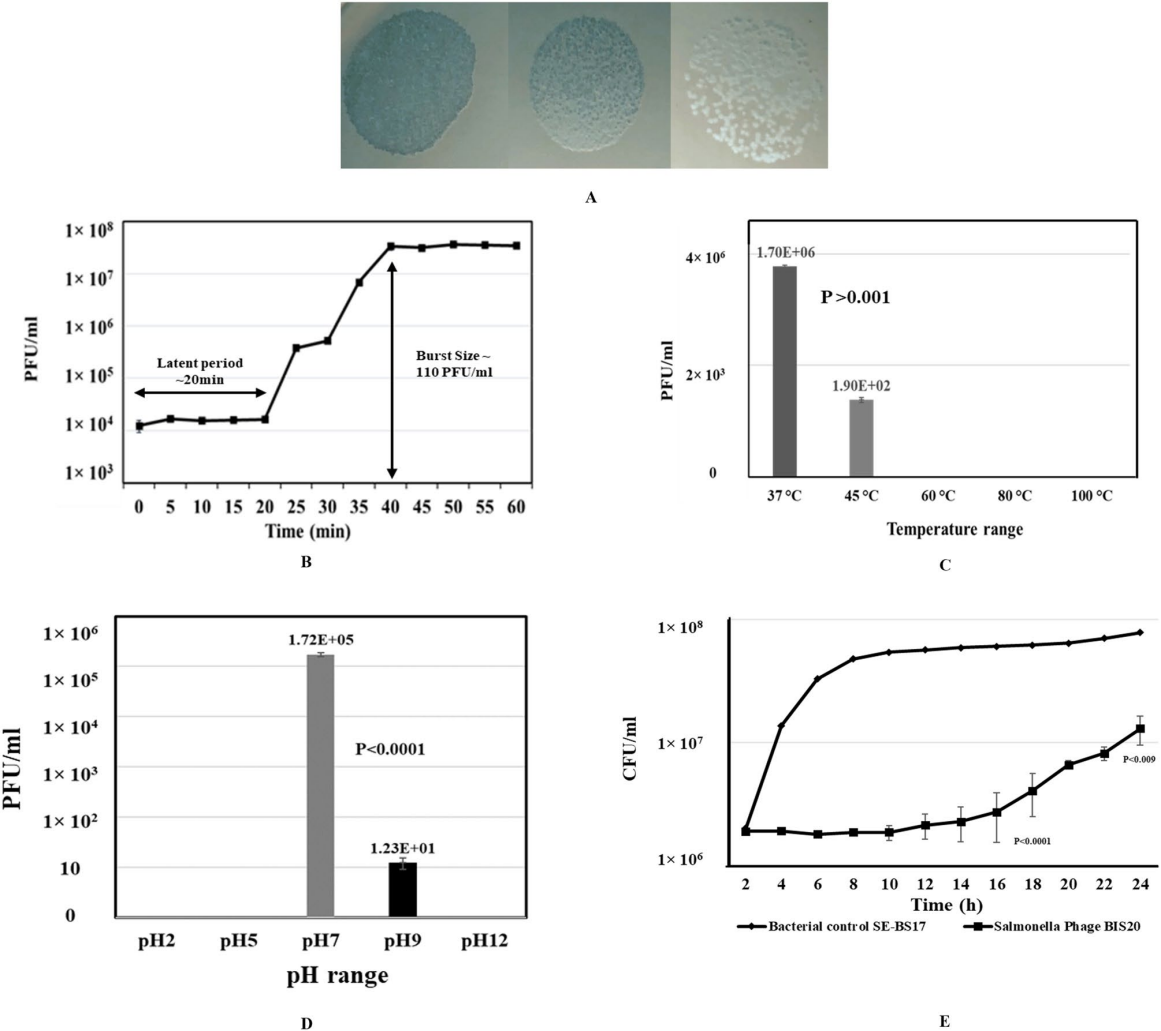
Physical Characterization of *Salmonella* phage BIS20: (**A**) BIS20 plaque morphology, turbid zones of lysis indicative of temperate nature of phage (**B**) Latent period and burst size of BIS20 determined by one step growth curve method (**C**) Temperature stability of BIS20; Phage was stable at 37 °C, barely survived at 45 °C, no plaques were observed at other temperatures tested. (**D**) BIS20 pH stability between 2 and 12 pH range; BIS20 grew best at pH7 and barely survived at pH 9 (**E**) Bacterial growth reduction assay; reading was monitored for 24 h, control; *Salmonella* Typhimurium (SE-BS17) in LB; SE-BS17 bacterial culture infected with *Salmonella* phage BIS20 at MOI1. All values are represented as average of triplicates.

*Salmonella* phage BIS20 was isolated from retail poultry tissue samples suspected of *Salmonella* infection using *Salmonella* Typhimurium strain SE-BS17 (Acc. # MZ503545) of poultry origin. BIS20 produced milky/turbid zones of lysis on SE-BS17 (clearing zone 0.9 mm) a characteristic that suggests it is a temperate phage later confirmed by presence of integration cassette in its genome (Fig. 1A)^19–21^. Host adsorption efficiency was 96% in 5 min after addition of BIS20 in SE-BS17 mid log phase culture (OD 0.35). The phage latent period as determined by a one-step growth curve, was 20 min with a burst size of 110 ± 7 PFU/cell (Fig. 1B, Supplementary Table S2). BIS20 host range was tested on 14 *Salmonella* strains. These strains were characterized up to subspecies level (materials and methods) and belonged to *Salmonella enterica* subspecies *enterica* however their serovars were not known. BIS20 produced clear zones of lysis on five strains only. Out of these five strains it grew best on SE-BS17, a *Salmonella* Typhimurium isolate as indicated by 16S rRNA sequencing (Supplementary Table S3). No lysis zones were produced on a strain of *S.* Typhi tested. Few other species of family *Enterobacteriaceae* tested by spot method were also not lysed. It is difficult to conclude the definitive host range of BIS20 as fewer strains were available for testing.

**Figure 2:**
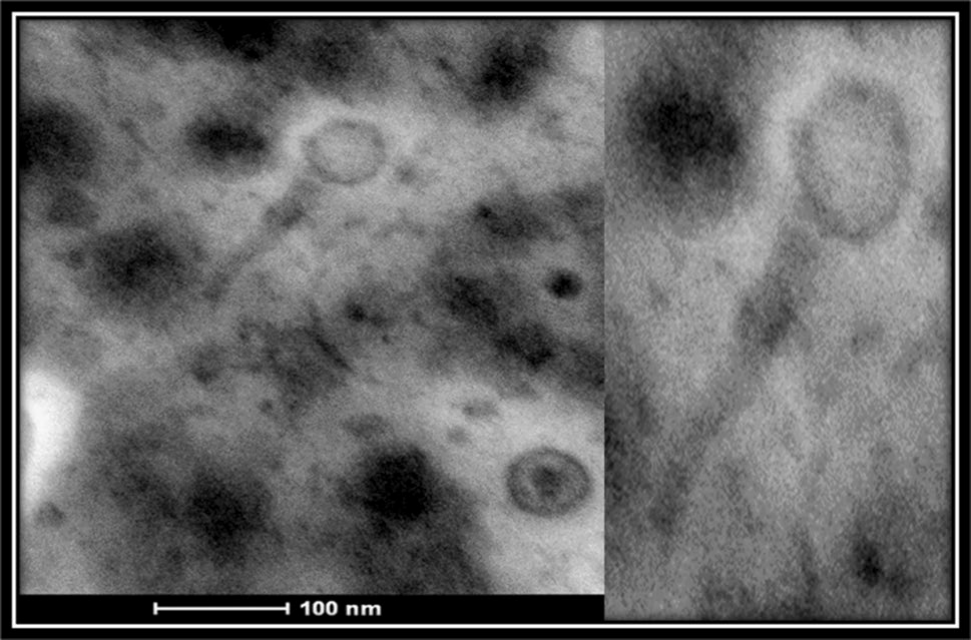
Transmission Electron Micrograph of *Salmonella* phage BIS20. Phage was stained with 1% uranyl acetate solution images were taken at an acceleration voltage of 80 kV. Scale bar represents 100 nm.

BIS20 produced maximum titer at 37 °C (1.7 × 10^6^); however, it was still able to infect at temperatures up to 45 °C (1.9 × 10^2^), albeit with a significant drop in titers (P < 0.001). BIS20 lost its infectivity after incubation at temperatures above 45 °C (e.g., 60 °C, 80 °C and 100 °C) as no plaques were observed at these temperatures (Fig. 1C). Highest titer of BIS20 was observed at pH7 (~ 1.72 × 10^5^ PFU/ml), whereas a significant (P < 0.001) drop in titer was observed at pH 9 (1.23 × 10^1^) after 1 h of incubation (Fig. 1D). No plaques / clearing zones were observed at pH 2, 5 and 12. Despite its temperate nature BIS20 was able to restrict growth of SE-BS17 up to 18 h post incubation at MOI 1 {BIS20 = 4.07 × 10^6^ , control (SE-BS17) = 6.18 × 10^7^} (P < 0.0001). The maximum growth of the SE-BS17 strain observed after 24 h of incubation with BIS20 at MOI 1 was 1.3 × 10^7^ CFU/ml which was significantly lower than bacteria only control (7.78 × 10^7^ CFU/ml) (P < 0.009) (Fig. 1E). Transmission electron microscope analysis indicated that BIS20 has a hexagonal head with a tail (Fig. 2).

### Genome characterization

**Figure 3:**
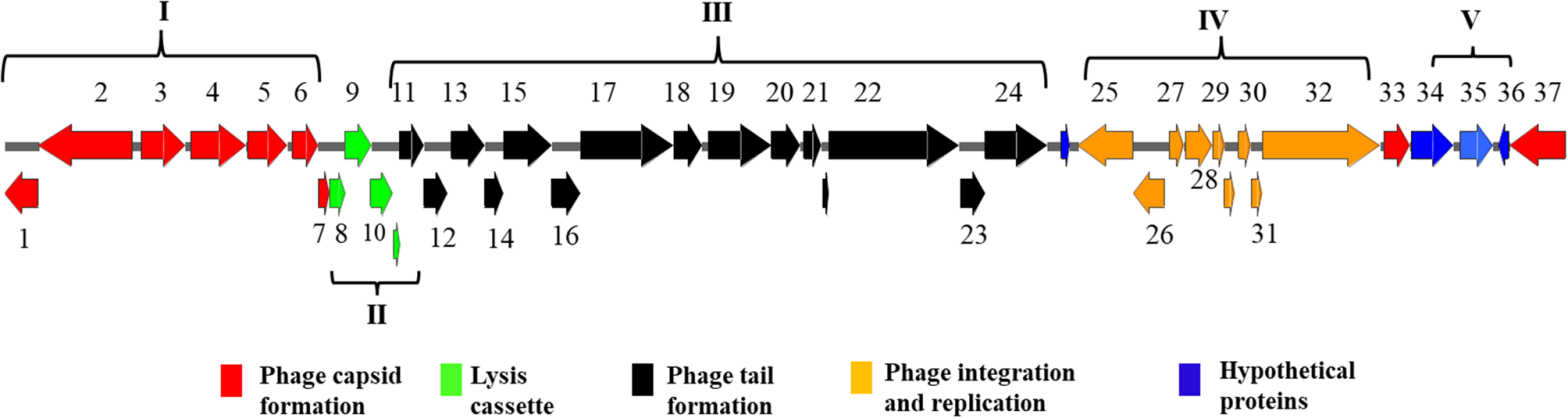
*Salmonella* phage BIS20 annotation map as generated by GeneMark.hmm, RAST and GAMOLA version 2 software. Different ORFs are color coded according to their putative functions. Numbers at each ORF correspond to their description in Supplementary Tables S4 and S5. For ease of description genome is divided in to five modules represented by Roman numerals and parentheses.

*Salmonella* phage BIS20 has a 29,477-base pair (bp) genome and encodes for 37 Open Reading Frames (ORFs). The genome map of BIS20 is given in Fig. 3. All ORFs are color coded according to their putative functions. The BIS20 genome has 53% GC content, well in alignment with its host strain (53.2%). Thirty-four ORFs started with an ATG start codon, one ORF coding for a replication endonuclease protein started with TTG (ORF 32), and two other ORFs started with GTG that include DNA damage inducible protein I (Din I) responsible for initiating SOS response (ORF 33), and a conserved hypothetical protein (ORF 1). The phage has a modular organization; each module is labelled by roman numerals in Fig. 3. Modules I and III include ORFs that encode structural proteins (ORFs colored red and black). Module I include ORFs involved in capsid formation, head stabilization and DNA translocation to empty capsids (Fig. 3, ORFs 1–7). These highly conserved proteins have homology with P2 family of *Enterobacteriaceae* temperate bacteriophages. ORFs in third module were involved in phage tail, tail sheath, tail fibers and base plate assembly (Fig. 3, ORFs 11–24). Majority of these proteins were conserved except for tail fiber domain containing protein (ORF 17) and tail fiber assembly protein (ORF 18). These two proteins of BIS20 have high homology with SEStP only (Supplementary Table S5) but not with phages. In addition, phage late control gene D protein, involved in lysis of cell and virion maturation (ORF24) was also present in this module. Module II includes host cell lysis enzymes and regulatory proteins (ORFs 7–10 colored green). BIS20 utilizes the usual conserved Lambda phage holin, lysozyme: spannin complex for bacterial wall lysis. All three components; holin, endolysin (lysozyme) and spannin show high gene and protein homology with *Salmonella enterica* and *Citrobacter *sp. prophages. These proteins carried putative conserved protein domains of *Escherichia* phage P2 lysis cassette. In addition, BIS20 features a GP25-like lysozyme, likely a structural component of base plate outer wedge with acidic lysozyme activity involved in phage entry into the host cell by binding with peptidoglycan (ORF14). Module IV has ORFs involved in phage integration (tyrosine recombinase (Xer C) /integrase) and lytic to lysogenic switching (*CI; immunity repressor*, *ApI; excionase* and *CII; Lysogeny regulation*) (ORFs 25–28; colored orange). Module V includes hypothetical proteins (ORFs colored blue). Two hypothetical proteins (ORF 35 and 36) were unique to BIS20. Detailed description of each ORF and their putative function with homology is given in Supplementary Tables S4 and S5.

BIS20 is a linear dsDNA phage as no contigs assembled across both ends of the phage genome Phage genome termini were analyzed by Phage Term software that utilizes two methods, a phage term method and Li’s method^22^. Analysis indicated presence of multiple termini on forward and reverse strands. Peaks localized 20 bases around the maximum indicated presence of preferred termini with terminal redundancy and apparition of partially circular permutations. The phage genome has direct terminal repeats or terminal redundancies. Such arrangement of termini protects linear DNA phages from DNA losses during replication. The packaging of such DNA takes place in to already made proheads that requires use of ATP dependent terminase activity to sort DNA concatemers (Supplementary Fig. S2).

### Phylogenetic analysis

#### BIS20 similarity with prophages

When BIS20 genome was compared in BLAST n the highest homology was shown by prophage sequences however genome coverage was low. As per ICTV recommendation VIRIDIC software was used to compare BIS20 genome with close phage and prophage homologs in BLASTn. VIRIDIC software is a tool to identify intergenomic similarities of bacteriophages and allow their classification into a genus or a separate species. According to VIRIDIC the BIS20 phage genome has highest homology of 87.7% with close homologs in NCBI, therefore it can be recognized as a separate species (nucleotide similarity less than 95%) however it can be placed in same genus as *Escherichia* virus 186 as nucleotide homology was more than 70%.

**Figure 4:**
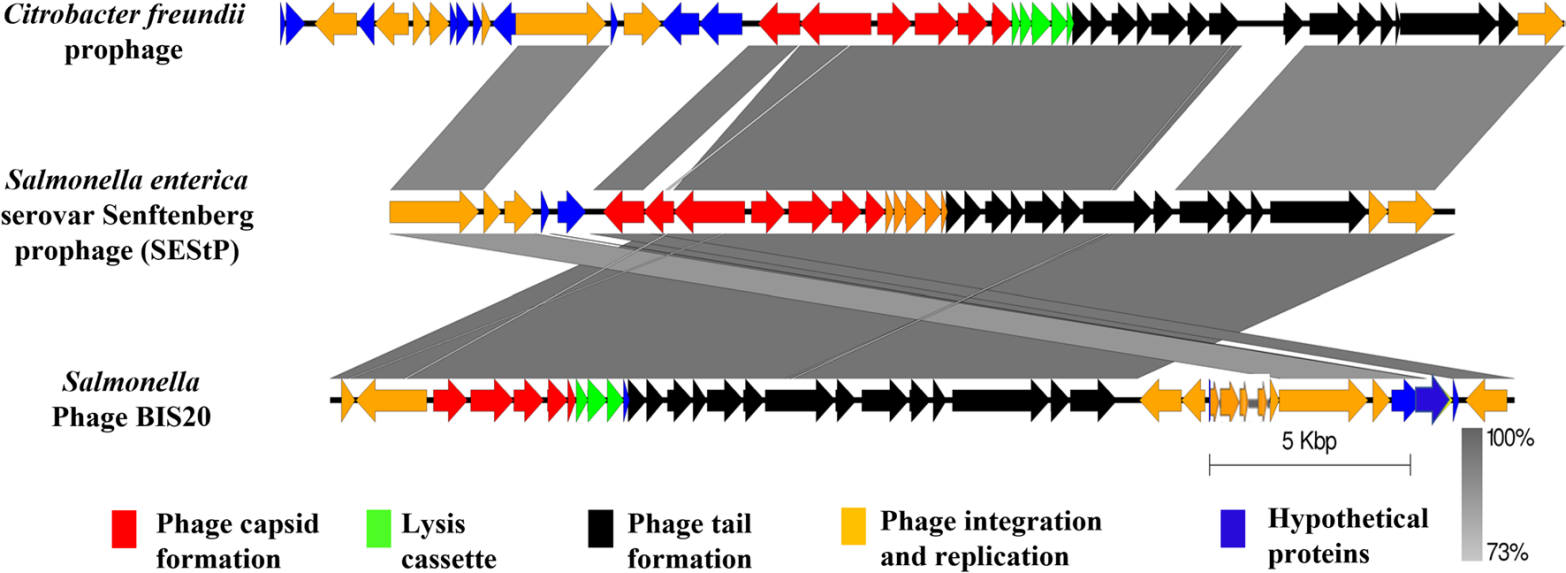
Genome comparison of BIS20 with closest prophage homologs *Salmonella enterica* serovar *Senftenberg* prophage (SEStP) and *Citrobacter freundii* prophage sequence. Various ORFs are color coded according to their putative function. (Scheme provided in Fig. 3).

BIS20 has highest similarity with *Salmonella enterica* sub species *enterica* serovar *Senftenberg* prophage (SEStP) sequence located in chromosome 1 (Acc. No; LS483465.1, sequence range 675,000–706,000) (Supplementary Table S5, Fig. 4), with only 87.7% nucleotide similarity. BLAST n results indicated that the proximal 21,368 bp of BIS20 has highest homology with SEStP (96%). This region has ORFs encoding for phage structural proteins. Remaining ~ 10 kb genome of BIS20 nucleotide sequence (22,078–29,477 bp) have varying degree of nucleotide homology (95–77%) with SEStP sequence whose ORFs encoded integration cassette, replication, and portal proteins among various other. Nucleotide sequence of two ORFs (26 and 35 Supplementary Table S4) ~ 710 bp each was missing from SEStP. ORF 26 encoded for phage repressor CI-C and ORF35 encoded a prophage / hypothetical protein having homology with prophage protein of *Klebsiella* pneumonieae (Acc no; SVY30080.1).

**Figure 5:**
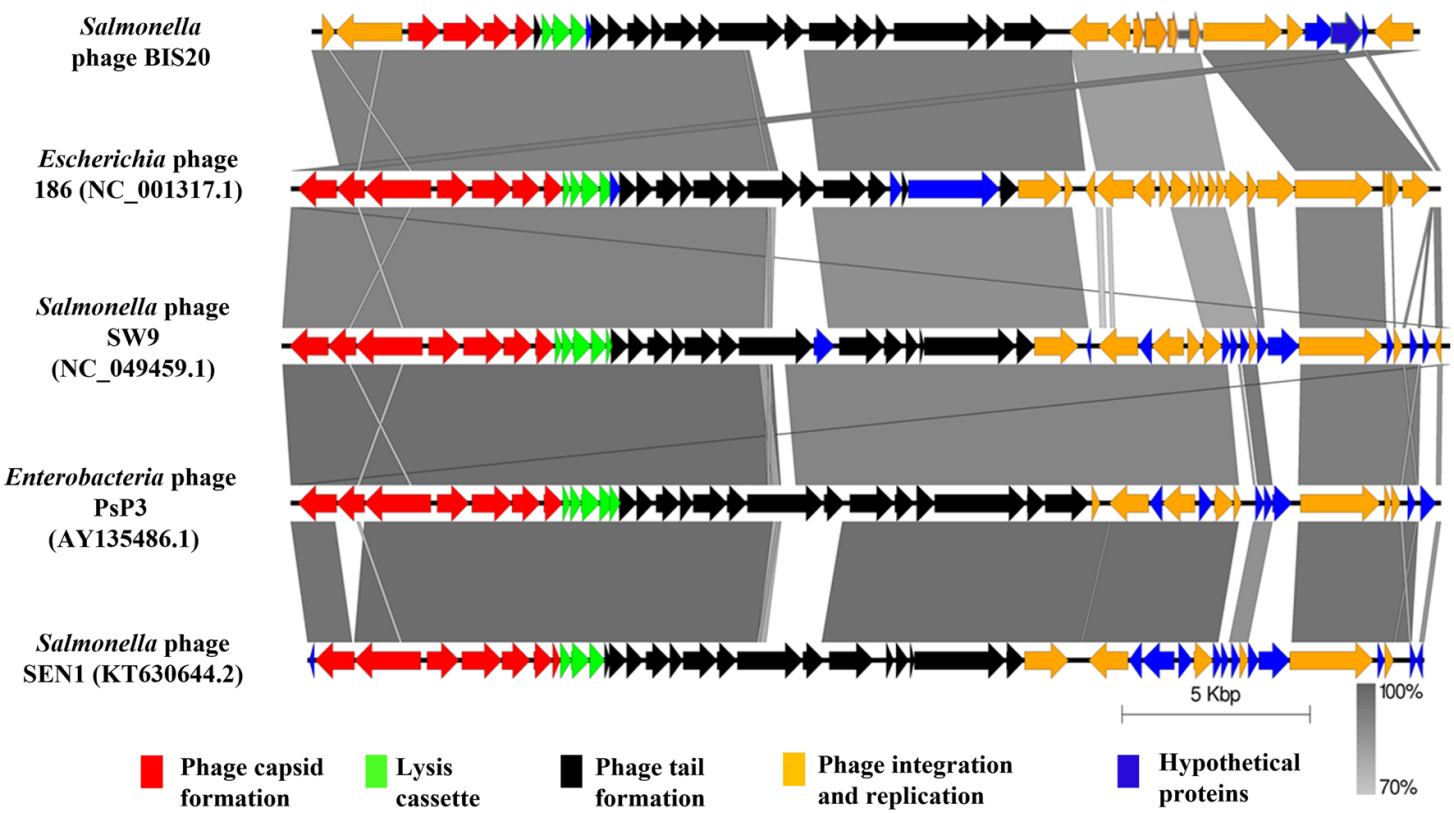
Genome comparison of BIS20 with closest bacteriophage homologs of genus *Eganvirus*. Various ORFs are color coded according to their putative function. (Scheme provided in Fig. 3).

**Figure 6:**
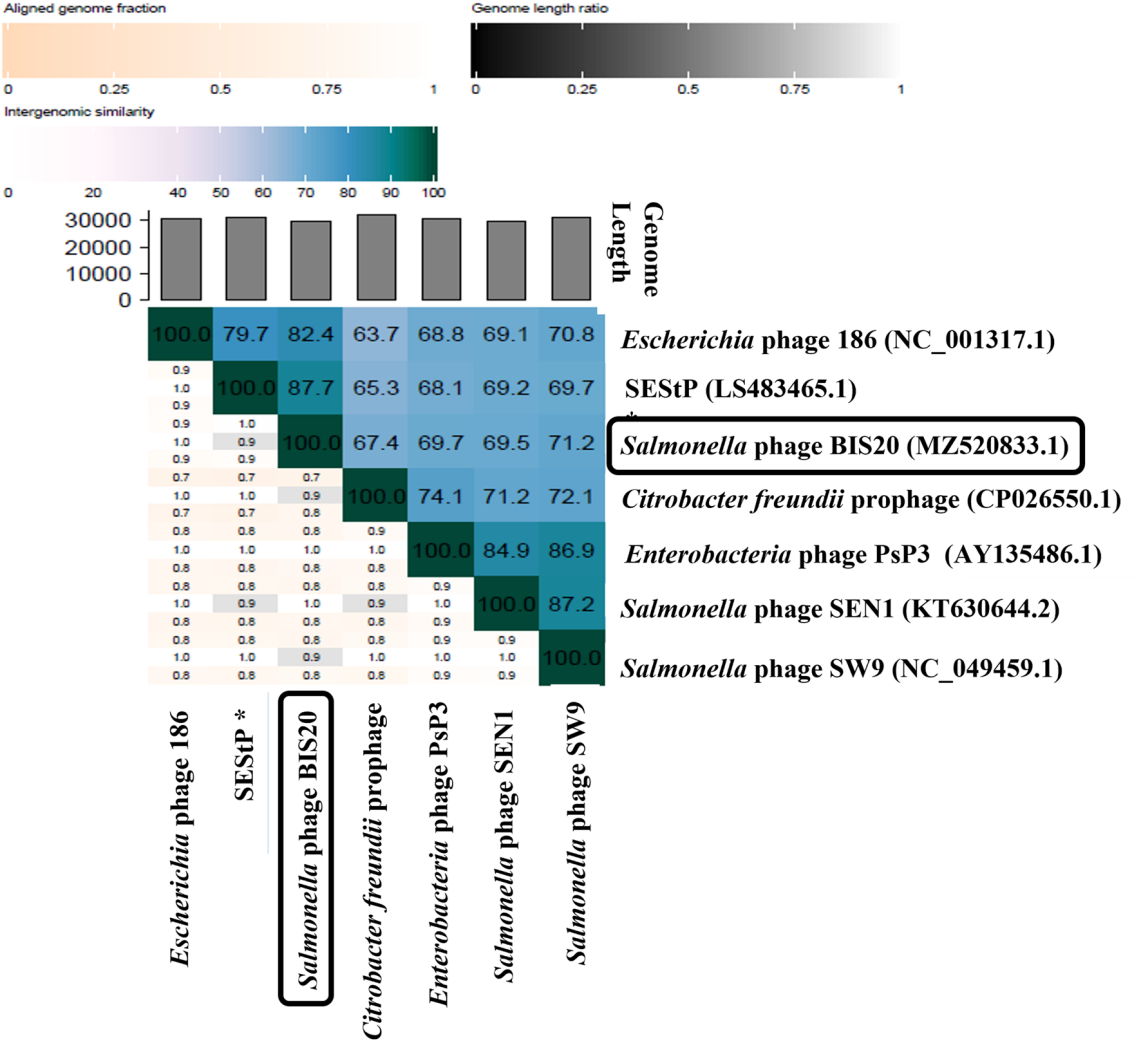
Heatmap generated by comparative genome analysis of *Salmonella* phage BIS20 with closest phage and prophage homologs in BLASTn using VIRIDIC software (http://rhea.icbm.uni-oldenburg.de/VIRIDIC/). Numbers in chart represent the homology percentage. *SEStP (*Salmonella enterica* sub species *enterica* serovar *Senftenberg* prophage).

Amino acid sequence of BIS20 ORFs has highest homology with SEStP as indicated by BLASTp, except for few proteins associated with base plate assembly and prophage induction. Overall BIS20 phage proteins are conserved and exhibit a mosaic pattern as they are found in different prophages of *Salmonella sp*. and *Citrobacter freundii* (Supplementary Table S4). SEStP is integrated exclusively between Siderophore interacting protein and mismatch specific DNA glycosylase of *Salmonella* chromosome. Interestingly just before SEStP in chromosome 1 (Acc. No LS483465.1) there is another prophage integrated in the same position which has no similarity with BIS20. The genome and protein similarities were also profound for *Citrobacter freundii* prophage (Gen. Bank Acc. No CP026550.1), with 67.7% nucleotide similarity (Figs. 4, 5). Integration site in *Citrobacter sp.* prophage matches, is between *OmpA* family protein and DNA binding transcription regulator. While these prophages retained the structural module of *Enterobacteriaceae* phage P2, they have acquired different proteins for interacting with specific hosts. The sequence of *Salmonella* phage BIS20 is deposited in NCBI with Acc. No MZ520833. Comparison of BIS20 genome sequence with these prophages is given in Figs. 4, 6.

#### BIS20 similarity with other bacteriophages

**Figure 7:**
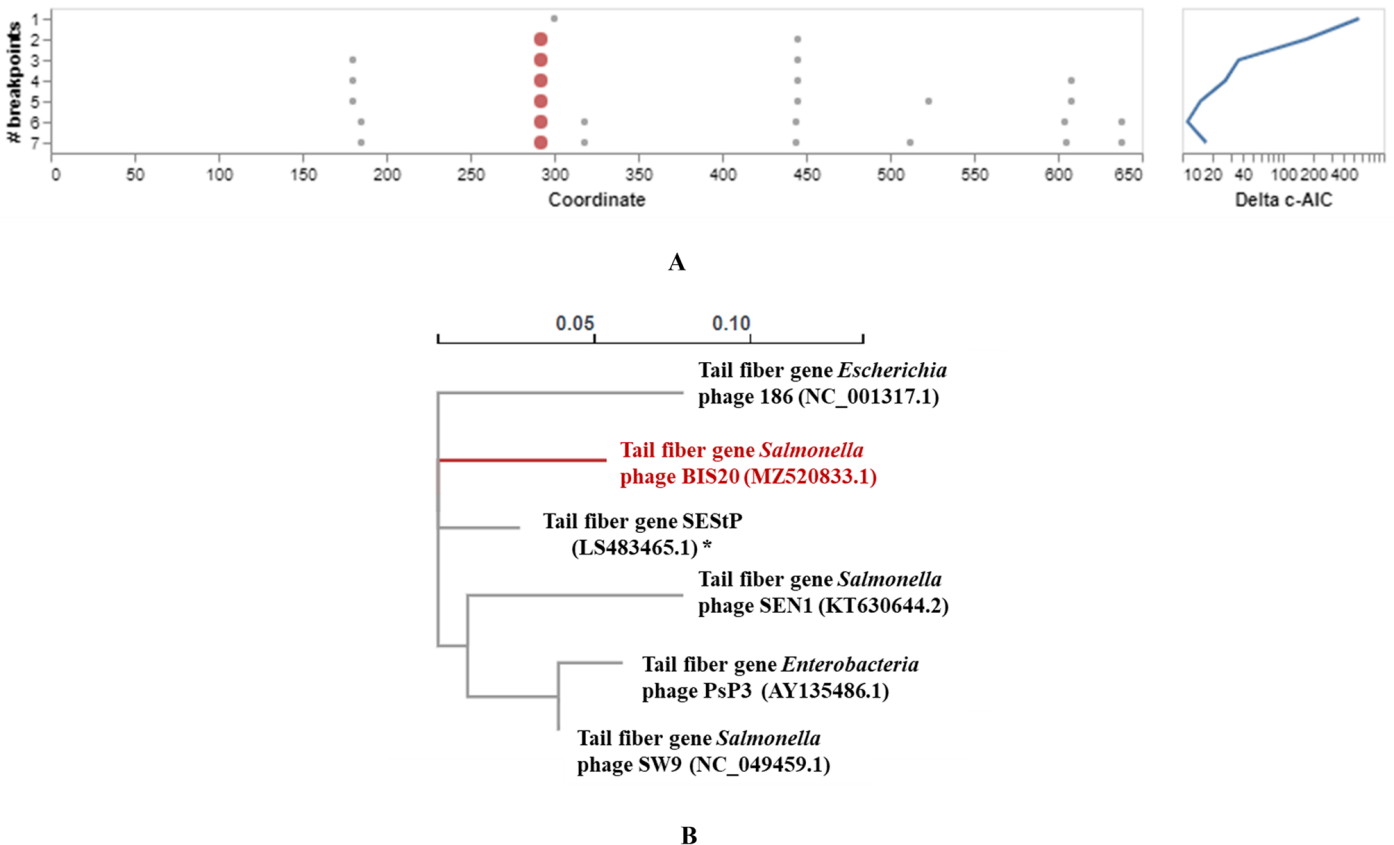
GARD analysis of the amino acid sequence of tail fiber gene with close homologs of BIS20. (**A**) Analysis identifies 8 breakpoints out of which the breakpoint at 300 position is the most probable breakpoint (represented with red dot). (**B**) Software generated phylogenetic tree made at coordinate range of 186–292 places BIS20 as a separate lineage.

The closest phage homologs of BIS20 were *Escherichia* phage 186 (Phage 186) (82.4%), *Salmonella* phage SW9 (SW9) (71.2%), *Enterobacteria* phage PsP3 (PsP3) (69.7%) and *Salmonella* phage SEN1(SEN1) (69.5%) as indicated by Easyfig and VIRIDIC analysis (Figs. 5, 6). These phages belong to genus *Eganvirus* of subfamily *Peduovirinae* (Supplementary Table S5, Figs. 5, 6). Interestingly the homology of all five phages had a conserved pattern. Genome of these phages was arranged in similar modules as BIS20. All of them exhibited 90–92% homology with BIS20 for the first 11 kb of genomic data having structural ORFs involved in capsid formation, lysis of host cell and part of tail formation. These ORFs were conserved and shared high sequence homology with each other and with BIS20. Followed by this region a ~ 1500 bp sequence of BIS20 encoding tail fiber domain and phage tail assembly protein (ORF17 and 18) was different in all phages and shared no sequence homology (Fig. 5). Interestingly, the initial 300 amino acids of tail fiber formation gene (TF) (ORF 17) were partially conserved in all phages (Supp. Figure S1) whereas remaining sequence has no homology. Amino acid sequence of TF from all phages was analyzed by GARD. It indicated presence of seven recombination hotspots of which one hotspot at amino acid 300 position was the most likely recombination site identified by eight breakpoint analysis (Fig. 7A). From this analysis it is presumed that active recombination at this site has enabled these phages to adapt to different hosts and expand their host range (Fig. 7B). It signifies the evolutionary contribution of such recombination events in giving rise to new phage species. We presume that one such event may have resulted in origin of BIS20 phage however it requires further experimental evaluation.

Further BIS20 nucleotide sequence of roughly 3500/4000 bp (Fig. 5) encoding tyrosine recombinase XerC and *CI* repressor (lytic to lysogenic switch) was absent from three phages SW9, SEN1 and PsP3. BIS20 integration cassette had similar arrangement to phage 186 but with less amino acid conservations. It consists of an integrase, a transcription regulator protein *CI* (whose expression leads to lysogenization, an *ApI* protein (similar to lambda *COX*) a putative excionase whose expression promotes lytic mode of replication and an additional protein *CII* that positively regulates lysogeny (Supplementary Tables S4 and S5). These BIS20 proteins had (98%, 62.44% (No nucleotide homology), 70.11% (No nucleotide homology), and 85.88% amino acid homology with Phage 186 respectively.

Another section of BIS20 genome approximately ~ 795 bp and 2151 bp was missing from *Escherichia* phage 186 and other three phages (SEN1, PsP3 and SW9) respectively (Fig. 5). This sequence encoded four ORFs in BIS20 (DNA damage inducible protein (ORF33), two hypothetical proteins (ORF 34 and 36) and a putative prophage protein (ORF35). In phage 186 only two ORFs 35 and 36 were missing whereas all four ORFs were missing from remaining three phages.

**Figure 8:**
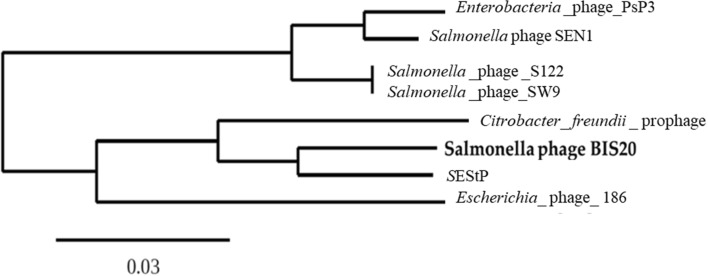
Phylogenetic tree of *Salmonella* phage BIS20 using whole-genome sequence of prophage and phage homologs in BLASTn. The maximum likelihood tree was generated using TreeDyn program and it provides a rough estimate of the relationships between related sequences in NCBI and *Salmonella* phage BIS20.

A phylogenetic tree was constructed for BIS20 using its close phage and prophage homologs in BLAST n. The modified alignment after removing the gaps was 18,096 nucleotides long. Best fit model on the data was found to be GTR + F + I. Maximum Likelihood tree was produced by the TreeDyn program. Two major clusters were found in the tree; *Salmonella* phages were found in both the clusters. BIS20 was found to be closest with *Salmonella enterica* serovar Senftenberg prophage genome. These two genomes were sister to *Citrobacter freundii* prophage genome. Basal to this cluster was *Escherichia* phage 186. The other cluster contained three *Salmonella* (SW9, SEN1, S122) phages and *Enterobacter* phage PsP3. *Salmonella* phage SW9 and S122 were identical in sequence in this region. According to this analysis BIS20 is classified as a separate species in genus *Eganvirus* (Fig. 8).

## Discussion

*S*. Typhimurium is associated with recent outbreaks of gastroenteritis worldwide due to consumption of partially cooked or raw poultry meat^1,2,23^. Temperate phages play a great role in host adaptability and pathogenicity. SE-BS17 used as host strain for BIS20 was characterized as *Salmonella enterica* subspecies *enterica* serovar Typhimurium by 16S rRNA sequencing. Although *S.* Typhimurium and *S.* Typhi are closely related to each other however BIS20 only infected *S.* Typhimurium, and not *S.* Typhi, this may indicate the recognition of only one host receptor by BIS20. Sustenance of only biological range of temperature and pH indicates that phage BIS20 may have adapted to its hosts’ physiology over the course of evolution^24^. *Salmonella enterica* harbors the richest pool of phages and prophages in its genome (roughly 9000 phage types)^25^ that have been extensively studied^6,26,27^. This study provides a detailed account of BIS20 physical properties and genome characterization. Nucleotide BLAST and VIRIDIC software analysis indicated that BIS20 is a new species of temperate phage closely related to members of genus *Eganvirus* of subfamily *Peduovirinae* family *Myoviridae*, this is in agreement with the fact that majority *Salmonella* temperate phages discovered so far; belong to family *Myoviridae*^28,29^*.* BIS20 has maximum homology with *Escherichia* phage 186 placed in the same genus and few prophage sequences in NCBI. The genome comprises of 53% GC content, well in alignment with its host strain (53.2%) indicating its evolutionary association with the host^30^. Presence of tRNAs within the phage genome is known to broaden host range by codon usage compensation, however, no tRNAs were identified in BIS20 genome^31^, moreover no DNA or RNA polymerases were present in genome indicating its dependence upon host.

The genome of BIS20 exhibited mosaicism as different ORFs were related to several different *Salmonella enterica* and *Citrobacter freundii* prophage sequences in the NCBI. The same phenomenon is indicated in a detailed comparative analysis of *Enterobacteriaceae* lambdoid phages previously^32–34^. Morphogenesis region is usually conserved in phages so was true for BIS20 whereas other genes have a mosaic arrangement probably due to previous crossovers and recombination events between phages. In BIS20 the genes responsible for binding with peptidoglycan; phage tail X protein, tail fiber and base plate assembly were less conserved and exhibited genetic diversity from both close prophage homologs (SEStP, *Citrobacter Sp.*).

BIS20 differs from its phage homologs by presence of unique long tail fiber gene (ORF17), tail fiber assembly genes (ORF18), as well as lytic to lysogenic switch proteins (ORF26-28). Interestingly these genes are different in all five members of *Eganvirus* genus. It is reported previously^35^ that over the course of evolution tail fiber genes of apparently unrelated phages can exchange segments resulting in phage mosaic genomes. This can lead to a change in host range of bacteriophages as well. A study published by Sandmeler^36^ indicated presence of DNA invertase and recombination site like sequences at both ends of tail fiber gene segments conferring host specificity. They also reported that 5ʹ end sequence of large tail fiber gene was conserved however 3ʹ end conferring host specificity is different among different phages. When we compared BIS20 and *Escherichia* phage tail fiber gene (ORF18); first 789 bp at 5ʹ end (~ 300 amino acids) have high homology (74%) however later 963 bp had ~ 49% nucleotide homology. Same was true for small tail fiber gene (ORF18, 49%). This observation indicates presence of a recombination hot spot in this region of tail fiber gene (ORF17) and same was found true by GARD analysis. Long tail fiber genes of three *Salmonella* specific bacteriophages SW9, S122 and SEN1 were also different from BIS20 although all of them infected *Salmonella* enterica serovars. The tail fiber gene of BIS20 was similar to the prophage homolog SEStP only. It is evolutionarily useful for phages to modify their proteins for interacting with host surface by site specific recombination and inversions. It suggests that BIS20 may have acquired these genes by similar mechanisms, GARD analysis indicated presence of 8 putative breakpoints in tail fiber genes of these phages out of which the best fit single breakpoint was at 300 amino acid position (Supp. Fig. S1). These two ORFS were present in *Salmonella* Senftenberg prophage (SEStP) sequence with 93 and 96% homology. BIS20 may have originated by recombination events between different phages and prophages from ancestral phage sequence by gene swapping^9,37^.

BIS20 has a tyrosine recombinase; a site-specific integrase for lytic to lysogenic switch which is like that found in SEStP and *Escherichia* Phage 186. However, the recombinases of other three phages SW9, PsP3 and SEN1 shared 90% amino acid homology with each other but only share ~ 53% homology with *Escherichia* phage 186 and BIS20. Phage 186 has a complicated integration cassette where instead of only *Int*, *C* (repressor) and *cox* (excionase lytic to lysogenic switch) protein of p2-phages; it has *Int*, *CI* (immunity repressor), *apI* (Excionase) and *CII* (regulation) proteins^38^. Both *CI* and *apI* genes of BIS20 have no nucleotide similarity with phage 186 however, protein sequence was 62% and 70% similar respectively. The exact mechanism of origin of this mosaicism is still debated. It is not clear whether it is product of random illegitimate recombination or due to specific recombination sites using DNA invertases and recombinases.

Temperate phages usually do not affect the ability of host to grow however BIS20 infection slowed down SE-BS17 growth to significant levels. Since the integration site for closest homolog SEStP is immediately before siderophore interacting protein, it may imply that integration event may have led to inactivation/ low efficiency of the siderophore interacting protein. Since this protein is crucial for iron uptake in the form of siderophores that play a critical role in bacterial growth^39^ its inactivation may have resulted in poor growth of the *Salmonella* Typhimurium strain SE-BS17. This ability of BIS20 may be utilized to explore its role in host pathogenesis. Our findings have identified BIS20 as a new phage species of genus *Eganvirus* that is an important link between *Salmonella* prophages and *Escherichia* phage 186. Further biological analysis may shed light on its evolutionary significance.

## Methods

### Bacterial strain isolation and characterization

In this study we used a *Salmonella enterica* serovar Typhimurium isolate of poultry origin for phage isolation. This isolate was previously isolated and characterized in Molecular Virology Labs (MVL) CUI, naming SE-BS17 (Gene Bank Acc No. MZ503545)^40^. Briefly sixty-five random poultry organ samples (Caecum, intestine, liver, and spleen) were collected from fourteen poultry farms in federal area supplying meat to retail poultry shops in Rawalpindi and Islamabad. The poultry samples were collected in sterile bags. Twenty-five grams of each poultry tissue sample (All tissue types pooled together) was homogenized to a fine paste using sterile surgical blades and pre enriched with 225 ml of 0.1% buffered peptone water (Oxoid). All samples were incubated overnight at 37 °C. After pre-enrichment 100 μl of broth culture was transferred to Selenite cystine broth (Oxoid) followed by incubation at 37 °C for 24 h. One loopful of enriched broths was streak plated onto Xylose Lysine Deoxycholate (XLD agar, Oxoid; CM0469) and incubated at 37 °C for 24 h. The plates were examined for typical colonies of *Salmonella*^41^. All isolates exhibiting typical *Salmonella* colonies on XLD plate were sub-cultured for biochemical characterization with API 20E strips (bio-Merieux; Ref No. 20100) as per manufacturers protocols.

Isolates identified as *Salmonella* (38 out of 65) by biochemical/numeric profile of API kit were further subjected to PCR amplification of 284 bp *InvA* gene for detection of *Salmonella* genus^42^ and *IroB* gene (606 bp) for the detection of subspecies (Supplementary Table S1). Out of these isolates one strain SE-BS17 was used for bacteriophage isolation. Serovar of SE-BS17 was confirmed by amplification and sequencing of 16S rRNA gene (Macrogen, South Korea) (Supplementary Table S1). The strain SE-BS17 was identified as *Salmonella* Typhimurium. Further it was tested for antibiotic sensitivity against common antibiotics used in poultry by Kirby-Bauer disc diffusion method^43^. SE-BS17 was tested for presence of beta lactamase (*bla*TEM-1, 643 bp)^44^, extended spectrum beta lactamase (*bla*CTX-M, 754 bp)^45^ and trimethoprim resistance gene *dfrA1* (474 bp)^46^ by PCR amplification. The PCR was carried out as per methods given in publications cited; briefly reaction was carried out in total 40 μl adding 8 μl master mix (Solis Biodyne, Cat No. 04-11-00S15) 29 μl sterile water 2 μl (10 pmol) each forward and reverse primers and 1 μl of template DNA (250 ng). Amplification was carried out in thermo-cycler (Thermo fisher) with following conditions; initial denaturation at 95 °C for 5 min; 35 cycles of denaturation at 95 °C for 30 s; different annealing temperatures as per GC content of each primer given in respective publications .Elongation was carried out at 72 °C for 45 s.; and final extension at 72 °C for 8 min. PCR products were subjected to gel electrophoresis with DNA ladder (Solis Biodyne, Cat No.07-12-0000S) as molecular marker (Supplementary Table S1).

### Bacteriophage isolation

Thirty-eight poultry samples (Caecum, intestine, liver, and spleen) previously tested positive for presence of different serovars of *Salmonella* as illustrated in “Bacteriophage isolation and characterization” were selected for phage isolation. The phage isolation was carried out as per protocols described by Duc^47^. Briefly 1 g of each of four tissue types in one sample were taken in a Petri plate and washed twice with Phosphate Buffered Saline (PBS, pH 7.2) to remove debris. These tissues were then triturated into fine mince by scalpel blade and mixed in 10 ml buffered peptone water and incubated at 37 °C for 24 h. Next day the media was centrifuged at 8000 rpm for 10 min to remove bacterial debris and supernatant was filtered through 0.22 μm syringe filters (CNW technologies). After filtration 100 µl overnight culture of SE-BS17 was mixed in filtrate supplemented with 10 mM CaCl_2_ and incubated at 37 °C for 24 h. Next day it was again centrifuged at 8000 rpm to remove bacterial cells and supernatant was filtered to obtain phage lysate. Phage lysate was stored at 4 °C until further use^48^. Next, phage lysate was serially diluted (1:10) in LB broth. One drop of each dilution was spotted on an LB agar plate carrying 200 µl of SE-BS17 overnight culture in soft agar (Agar overlay method). All samples that produced individual plaques or lysis zones on SE-BS17 *Salmonella* strain after 24 h of incubation at 37 °C were considered positive and selected for further purification.

### Plaque purification and phage stock preparation

Double layer agar overlay method was used for further plaque purification of *Salmonella* phage BIS20 (referred to as BIS20). One plaque from the phage-agar overlay plate was pierced using a tip-cut standard blue tip and the agar plug was ejected in 1 ml PBS (pH 7.2). The tubes were agitated for 1 h and then centrifuged at 10,000 rpm at 4 °C for 10 min. Supernatant was filtered through 0.22 μm syringe filters and kept at 4 °C until further use. The process was repeated twice to minimize the risk of contamination. For large scale preparation and amplification of BIS20; 1 L of LB medium (Oxoid) was inoculated (1: 10) with overnight grown culture of SE-BS17 and incubated at 37 °C with shaking till log phase (OD_600_ 0.55) and then infected with phage at Multiplicity of Infection (MOI) 1. Infected cultures were incubated overnight as described previously^49^. After overnight incubation, the culture was spun at 6,000 rpm for 10 min at 4 °C. Supernatant was filtered through 0.22 μm filter and stored at 4 °C. Phages were further concentrated by Polyethylene Glycol 8000 (PEG) (Sigma Aldrich Cat. No; 1546605) precipitation as reported elsewhere^50^. Briefly, 10% w/v PEG was added to 1 L phage lysate and was kept without shaking overnight at 4 °C. Samples were centrifuged at 8,000 rpm at 4 °C (centrifuge; Helmer, Germany) for 40 min. Supernatant was decanted and the precipitate was dissolved in 2 ml Phosphate Buffered Saline (PBS) pH7.2 (137 mM NaCl, 2.7 mM KCl, 8 mM Na_2_HPO_4_, and 2 mM KH_2_PO). The PEG-Enriched lysate was filtered through a 0.22 μm filter (HYSNY25022, Nylon syringe filters) and stored at 4 °C. The final phage titer was determined by the agar overlay spot method of serial two-fold dilutions of phage lysate as described in “Genome characterization”.

### Phage host range

Host range of BIS20 was determined by its ability to produce clear zones of lysis on each strain by spot method (10 µl drops of BIS20 stock, 1.8 × 10^5^ pfu/ml) using soft agar overlays^51^. A list of *Salmonella enterica* and other isolates used for host range determination is given in Supplementary Table S2. The host range was determined as per protocol used by Mazzocco. Briefly, 200 µl of overnight culture of each strain was mixed with 2.5 ml of soft agar (0.5% agar dissolved in LB) in 5 ml Kimax glass tubes (Sigma Aldrich Cat. No; Z255122). The mixture was then poured on to LB agar (Oxoid) plates and allowed to solidify for 20 min. Filtered phage lysate drops of BIS20 having titer 1.8 × 10^5^ pfu/ml (10 µl) were placed on top of the seeded soft agar plates without dilutions. Presence of clear / turbid lysis zones was examined after 24 h. Bacterial strains were categorized as sensitive (+ ; lysis) or resistant (− ; no-lysis) based on presence or absence of clear/ turbid lysis zones.

### One step growth curve

Burst size and latent period of BIS20 were determined by the one step growth curve method with some modification to the method previously reported^52^. Briefly, 40 ml LB broth was inoculated with *Salmonella* strain SE-BS17 overnight culture (1:100) and incubated at 37 °C with shaking until the culture reached log phase (O.D._600_ 0.5) (2 × 10^7^ CFU/ml). At log phase the culture was infected with BIS20 at MOI 1(3.23 × 10^5^ pfu/ml). The culture was left stationary for 15 min at 37 °C to allow phage adsorption and then centrifuged at 11,000 rpm for 1 min. The supernatant was transferred into new tubes and later titrated for enumeration of un-adsorbed phage particles. Cell pellet was washed three times with 1 ml fresh LB medium. After three washes the pellet was suspended in 30 ml fresh LB and allowed to grow at 37 °C for 1 h. One ml aliquots were collected from growing BIS20 infected SE-BS17 culture at 5 min interval for 60 min. The aliquots were immediately centrifuged, filtered, and then titrated after dilutions using agar overlay method. Burst size was calculated using a formula given in Supplementary Table S3^19^.

### Phage stability assay

The stability of BIS20 was tested by incubating fixed number of phages (1.8 × 10^5^pfu/ml) at different temperatures and pH values as described previously^53^. Briefly, for assessment of thermal stability, 1.8 × 10^5^ pfu of BIS20 were suspended in 1 ml PBS in triplicates and incubated at 37 °C, 60 °C, 80 °C and 100 °C respectively for 1 h. To assess BIS20 ability to sustain different pH values PBS with different pH values (2, 5, 7, 9, and 12) was prepared. pH was adjusted by either 6 N HCl or 6 M NaOH. Phages were suspended into this buffer to a final concentration of 1.8 × 10^5^ pfu. These pH solutions were incubated at 37 °C for 1 h and then surviving phages were calculated immediately by double layer agar overlay platting method as described previously. The results are presented as average of triplicates.

### Bacterial growth reduction assay

To determine the ability of BIS20 to lyse *Salmonella* Typhimurium (SE-BS17), bacterial growth reduction was quantified as described previously^54^. Briefly, an overnight culture of SE-BS17 was diluted in 40 ml LB broth in four 100 ml glass flasks to a final concentration of 1.8 × 10^5^ CFU/ml. Three flasks were inoculated with BIS20 at MOI 1. Fourth flask was used as bacteria only control with no phage added. After every 2 h, one ml culture was taken out of each flask using sterile disposable plastic pipette and its optical density (OD_600_) was measured using a spectrophotometer (Hinotek, 721-100G). A standard curve was plotted to determine bacterial colony forming units at various optical density values by dilution plating. This curve was used to determine the CFU/ml at corresponding optical density at 600 nm for growth reduction assay. Results are presented as average of triplicates.

### Electron microscopy

For negative staining, 5 µL of PEG purified phage sample (~ 6 × 10^6^ pfu/ml) was applied onto glow-discharged carbon coated grids and incubated for five minutes at room temperature. Grids were washed with 5 µL of deionized water before incubating for 2 min with a 1% uranyl acetate solution. Electron micrographs were taken using a Phillips CM-10 microscope at the Manawatu Microscopy and Imaging Centre (MMIC, Massey University, Palmerston North, New Zealand)^55^.

### Sequence and bioinformatic analysis of BIS20

Bacteriophage DNA was isolated using phage DNA isolation kit of Norgen biotek Corp. (Cat # 46800) as per manufacturer’s protocol^56^. Phage genome sequencing was performed using the Illumina MiSeq platform at Massey Genome Service (Massey University, Palmerston North, New Zealand). Briefly, the DNA library was prepared using the Illumina Nextera™ XT library preparation kit_V2 (Illumina, San Diego, CA, USA). Phage genomic DNA was enzymatically sheared into random fragments. Barcoded Illumina adapters were added onto each end of the fragments during enrichment PCR. Libraries were QC checked with a PerkinElmer GX Touch HT Instrument using the labchip DNA high sensitivity and a Quant-iT dsDNA HS assay using a PerkinElmer Victor Plate Reader. Libraries were pooled before loading onto the Illumina MiSeq™ run. Libraries were sequenced using Illumina MiSeq Micro 300 cycle kit V1 (Illumina, San Diego, CA, USA) and generated 2 × 150 base paired end reads. PhiX control reads, and adapter sequences were removed using FASTQMCF^37,38^. Sequence reads then trimmed at an error probability of 0.01 (Pared score of Q20). Sequence data was tested for quality control (QC) using Fast QC (Version 0.11.5 released), Fast Q screen and Solexa QA +  + (37). Contigs were assembled using Artimis software^59^. The assembled genome was annotated using GAMOLA version 2, RAST (The server is freely available at http://RAST.nmpdr.org) and GeneMark.hmm (http://opal.biology.gatech. edu/GeneMark/gmhmmp.cgi) software. ORFs and putative genes were identified by HHpred (available at https://toolkit.tuebingen.mpg.de/tools/hhpred) and RAST. Individual ORFs protein sequence data from BIS20 was analyzed by NACBI, BLASTp as well as HHpred. software. Phage is a linear dsDNA phage as no contigs assembled across both ends of the phage genome however the sequence of phage termini was analyzed using Phage Term software. It requires Individual BIS20 FASTAQ reads and a FASTA file of assembled genome. The PhageTerm software is freely available at pasture web server (https://galaxy.pasteur.fr/)^22^. The report generated by this software is included in Supplementary material file (Supplementary Fig. S2). The genome sequence of BIS20 is added to NCBI database with name “*Salmonella* phage BIS20” as per ICTV recommendations of naming a phage^60^ under Acc. No. MZ520833.

### Phylogenetic analysis

For genome wide comparison of BIS20 with related phage and prophage sequences VIRIDIC software was used. VIRIDIC applies the traditional algorithm recommended by International Committee on Taxonomy of Viruses (ICTV), subcommittee of Bacterial and Archaeal Viruses (BAVS) to calculate phage intergenomic similarities. VIRIDIC software has highest agreement with BLASTn. Genome comparison figures were generated using Easyfig software which is freely available (under a GPL license) for download at https://mjsull.github.io/Easyfig/^61^.

Since a simplified phylogenetic tree cannot adequately explain evolution of homologous sequences that are governed by gene conversion and recombination, we used multiple sequence alignment of tail fiber genes to search recombination breakpoints with high degree of quantitative support for their existence^62^. Recombination hotspots were identified by analysis through GARD software which is freely available at Datamonkey adaptive evolution server (https://www.datamonkey.org/gard).

To construct a traditional phylogenetic tree seven phage/prophage sequences having sequence homology with BIS20 were downloaded from NCBI. These sequences were aligned with BIS20 genome using MAFFT alignment^63^ implemented in Geneious (v.8.1.9)^64^. Alignment was trimmed from both ends and the gapped sequences / insertion and deletions were visually located and removed from the alignment. The alignments in Phylip format were imported in IQtree^65^ online version is available at http://iqtree.cibiv.univie.ac.at// to reconstruct a Maximum Likelihood tree based on the best fit model as implemented in the IQTree^66^. IQTree implements Model Finder^67^ to calculate best fit model on the data. The trees in Newick format were refined in the online TreeDyn tool^68^ available at http://www.phylogeny.fr/one_task.cgi?task_type=treedyn and downloaded in pdf format.

### Statistical analysis

Statistical analyses were performed with Origin 2019 (Origin Lab, Northampton, MA). The statistical significance was determined using two sample t test for specific comparisons. Statistical significance was reached at p < 0.05.

## Supplementary Information

Supplementary Information.

## Data Availability

The genome sequence of *Salmonella* phage BIS20 with the same name is deposited in NCBI GenBank with Acc. No MZ520833. Data is publicly available now.
