# Supplementary material for: Phenotypic characterization and genome analysis of a novel Salmonella Typhimurium phage having unique tail fiber genes
Source: Sci Rep. 2022 Apr 6;12:5732. doi: 10.1038/s41598-022-09733-5 (PMC8986868; doi:10.1038/s41598-022-09733-5)
Supplement: Supplementary file 1 — Supplementary Information. [file 41598_2022_9733_MOESM1_ESM.pdf]

Supplementary table 1; Oligonucleotides used for SE-BS17 characterization

Primer code	Target Gene	Oligonucleotide Sequence (5´-3´)	Size (bp)
Salmonella identification	*invA*	F’ TCATCGCACCGTCAAAGGAACC<br>R’ GTGAAATTATCGCCACGTTCGG GGCAA	284
	iroB	F’ TGCGTATTCTGTTTGTCGGTCC<br>R’ TACGTTCCCACCATTCTTCCC	606
	fliC	F’ TATGCCGCTACATATGATGAG<br>R’ TTAACGCAGTAAAGAGAG	495
Beta-Lactams	*blaTEM-1*	F’ CAGCGGTAAGATCCTTGAGA<br>R’ ACTCCCCGTCGTGTAGATAA	643
	blaCTX-M	F’ CGCTGTTGTTAGGAAGTGTG<br>R’ GGCTGGGTGAAGTAAGTGAC	754
Trimethoprim resistance gene	*dfrA1*	F’ GTGAAACTATCACTAATGG<br>R’ TTAACCCTTTTGCCAGATTT	474bp
16S rRNA	PCR primers	27F 5' AGA GTT TGA TCM TGG CTC AG 3<br>1492R 5' TAC GGY TAC CTT GTT ACG ACT T 3'	Macrogen Korea
	Sequencing Primers	785F 5' GGA TTA GAT ACC CTG GTA 3'<br>907R 5' CCG TCA ATT CMT TTR AGT TT 3'	

Supplementary table 2; Calculations for burst size of *Salmonella* phage BIS20

**Phage ID**	**Total applied phages (A)**	**Free phages (B)**	**Phages after burst (C)**	**Burst size (D/E)***
**Replicate 1**	$3.23x10^5$	$2.2x10^4$	$3.12x10^7$	103
**Replicate 2**	$3.23x10^5$	$1.4x10^4$	$3.33x10^7$	104
**Replicate 3**	$3.08x10^5$	$1.68x10^4$	$4x10^7$	127
**Average Burst Size**	-	-	-	~110 particles /cell

*Formula: Burst size = New phages released (D) / Number of infecting phages (E)

= **C-B/ A-B = D/E**

Supplementary table 3; Host range of *Salmonella* phage BIS20

S. #	Isolate ID	Source	*Salmonella* Isolate	BIS20 sensitivity
1	SE-BS17	Poultry	*Salmonella enterica* Typhimurium	+++*
2	SE-BS19	Poultry	*Salmonella enterica*	----- ǂ
3	SE-BS18	Poultry	*Salmonella enterica*	+++
4	SE-BS10	Poultry	*Salmonella enterica*	+++
5	SE-BS23	Poultry	*Salmonella enterica*	------
6	SE-BS9	Poultry	*Salmonella enterica*	+++
7	SE-BS32	Poultry	*Salmonella enterica*	-----
8	SE-BS20	Poultry	*Salmonella enterica*	-----
9	SE-BS22	Poultry	*Salmonella enterica*	++
10	SE-BS7	Poultry	*Salmonella enterica*	-----
11	SE-BS21	Poultry	*Salmonella enterica* Gallinarum	-----
12	SE-BS13	Poultry	*Salmonella enterica* Gallinarum	------
13	SE-BS12	Poultry	*Salmonella enterica* Gallinarum	------
14	Kp-JMSS15a	Human	*Klebsiella pneumoniea* (OK086689)	------
15	S.aureus-JMSS15b	Human	*Staphylococcus aureus* (OK086690)	------
16	E.fergusonii-QZJM25	Poultry	*Escherichia fergusonii*	------
17	EF-JMSS09	Human	*Enterococcus facialis* (MZ496438)	------
18	S16	Human	*Salmonella* Typhi	------
19	SP2	Human	*Streptococcus pyogenes*	------
21	Eh-SSJM01	Human	*Enterobacter hormaechei* (OK086761)	------
22	AB-ZAJM6	Human	*Acinetobacter baumannii* (MZ496431)	------

*Produced clear zones of lysis on this strain= Sensitive

ǂ Produced no clearing zones= resistant

Supplementary table 4 ; BIS20 predicted Open Reading Frames (ORFs) and similarity to the closest BLAST hits (Blast n)

ORF	Start	End	Putative Function	Total AA (DNA bp)	Query coverage	Percentage identity	GB Ac. NO	Organism
1	284	619	Conserved Hypothetical protein	112<br>339 bp	100 %	97.74 %	CP042441.1	*Salmonella enterica serovar* Senftenberg
2	637	2404	Terminase ATPase subunit	589AA<br>1770 bp	100 %<br>100 %	97.74 %<br>97.74 %	CP026550.1<br>LS483465.1	*Citrobacter* species<br>*Salmonella enterica serovar* Senftenberg
3	2572	3426	Phage Capsid Scaffolding Protein	284AA<br>855 bp	100 %	98.83	CP032194.1	*Salmonella enterica serovar* Senftenberg
4	3503	4570	Phage major Capsid Protein P2 family	355AA<br>1068 bp	99 %<br>100 %	97.55%<br>97.38%	CP010140.1<br>CP014356.1	*E. coli*<br>*Salmonella enterica*. Typhimurium
5	4574	5323	Terminase endonuclease subunit	249 AA<br>750 bp	100 %<br>100 %	98 .13 %<br>98 %	CP028169.1<br>CP026550.1	*Salmonella enterica*<br>*Citrobacter Freundii*
6	5416	5922	Phage capsid completion protein	168 AA<br>507 bp	100%	98.42%<br>97.63 %<br>96.84 %	CP056647.1<br>CP018976.1<br>CP074257.1	*Citrobacter* Sp<br>*E.coli*<br>*Salmonella*
7	5922	6125	Phage tail X protein	67 AA<br>204 bp	100 %	96.6%<br>93.79%<br>90.96%	CP056647.1<br>CP018976.1<br>CP006602.1	*Citrobacter*<br>*E.coli*<br>*Salmonella*
8	6132	6386	Phage holin family 2	98 AA<br>255 bp	100%	98.82%<br>98.43%<br>98.43%	CP056647.1<br>LS992175.1<br>CP006602.1	*Citrobacter*. Sp<br>*E.coli*<br>*Salmonella enterica*
9	6412	6909	Lysozyme	165 AA<br>498 bp	100%	97.59%	CP056647.1	*Citrobacter*. Sp
10	6960	7313	Phage lysis regulatory protein; Spannin	118 AA<br>354 bp	100 %	99.72%	CP026550.1	*Citrobacter*. Sp

11	7427	7894	Phage tail completion protein	155 AA<br>468 bp	100 %	98.50%	CP026550.1	*Citrobacter*. Sp
12	7886	8336	phage virion morphogenesis protein_13	149AA<br>450 bp	100%	98.67%	CP026550.1	*Citrobacter* Sp.
13	8405	9046	Phage base plate assembly protein	213 AA<br>642 bp	100%	97.66%<br>97.04%	CP056647.1<br>LS483465.1	*Citrobacter* Sp<br>*Salmonella enterica serovar* Senftenberg
14	9088	9360	Gene 25 like lysozyme	115 AA<br>273 bp	100 %	99.63 %<br>99.27 %	CP056647.1<br>CP019404.1	*Citrobacter*. Sp<br>*Salmonella enterica serovar* Bardo
15	9397	10305	Baseplate assembly protein	302 AA<br>909 bp	100 %	93.73%<br>92.96%	CP032184.1<br>CP028169.1	*Citrobacter Freundii*<br>*Salmonella enterica*
16	10316	10726	Prophage function phage tail protein I	177 AA<br>411 bp	100 %	97.08 %	LS483465.1	*Salmonella enterica serovar* Senftenberg
17	10839	12590	Tail fiber domain containing protein	583 AA<br>1752 bp	100 %	94.52 %	CP019201.1	*Salmonella enterica serovar* Muenster
18	12593	13117	Tail fiber assembly protein	174 AA<br>525 bp	100 %	96.19%	LS483465.1	*Salmonella enterica serovar* Senftenberg
19	13248	14426	Tail sheath protein	392 AA<br>1179 bp	100 %	97.29%<br>97.20%	CP056653.1<br>CP028169.1	*Citrobacter Freundii*<br>*Salmonella enterica*
20	14442	14963	Phage major tail tube protein	173 AA<br>522 bp	100 %	97.5%<br>96. 93%	CP032184.1<br>NC_001317.1	*Citrobacter Freundii*<br>*E coli* phage 1*86*
21	15027	15362	Hypothetical protein (Putative tail assembly chaperon)	111 AA<br>336 bp	100 %	98.81%<br>97.62%	CP056653.1<br>NC_001317.1	*Citrobacter Freundii*<br>*E. coli* phage 186
22	15507	17948	Phage tail tape measure protein	813 AA<br>2442 bp	100 %	97.99%<br>96.15 %	CP032184.1<br>CP028169.1	*Citrobacter Freundii*<br>*Salmonella enterica*
23	17962	18447	Bacteriophage P2 GPU, tail assembly protein	161 AA<br>486 bp	100 %	97.22%	CP053865.1	*Salmonella enterica serovar* Typhimurium
24	18444	19613	Phage late control gene D protein	389 AA<br>1170 bp	100 %<br>98 %	95.90%<br>96.34%	CP032184.1<br>CP039713.1	*Citrobacter Freundii*<br>*Salmonella enterica*
25	20167	21210	Tyrosine recombinase	347 AA	100 %	98.95 %	CP032184.1	*Citrobacter Freundii*

			Xer C, integrase	1044 bp	99 %	96.35 %	CP030211.1	*Salmonella enterica*
26	21210	21797	Phage CI-C repressor, lytic-lysogenic switch	195 AA<br>588 bp	100 %<br>99 %	99.01 %<br>94.88 %	CP032184.1<br>LR596811.1	*Citrobacter Freundii*<br>*Klebsiella Pneumoniae*
27	21901	22164	*ApI*, Hypothetical Protein Putative Excisionase	87 AA<br>264 bp	100 %	99.49 %	CP032184.1	*Citrobacter Freundii*
28	22195	22704	Hypothetical Protein, CII bacteriophage 186, putative DNA binding protein involved in lysogeny	169 AA<br>510 bp	100 %	98.48%<br>92.94 %	CP032184.1<br>CP030211.1	*Citrobacter Freundii*<br>*Salmonella enterica*
29	22712	22939	Protein Fil	75 AA<br>228 bp	100 %	97.37 %	LS483465.1	*Salmonella enterica serovar* Senftenberg
30	23193	23426	DUF 2732 unknown function protein	77 AA<br>234 bp	100 %	97.44%<br>97.01%	CP044098.1<br>CP025280.1	*Citrobacter* Sp<br>*Salmonella enterica serovar Brandenburg*
31	23426	23647	DSKa like Zinc finger like domain protein, phage conjugal plasmid C4	73 AA<br>222 bp	100 %	99.02 %<br>98.04	CP058014.1<br>CP056647.1	*E coli*<br>*Citrobacter* Sp.
32	23649	25865	Replication endonuclease	738 AA<br>2217 bp	99%<br>96%	96.55 %<br>95.61 %	CP044098.1<br>CP025280.1	*Citrobacter* Sp.<br>*Salmonella enterica*
33	25983	26423	DNA damage inducible protein, SOS operon	146 AA<br>441 bp	100%	99.09	CP044098.1	*Citrobacter* Sp
34	26453	27238	Hypothetical Protein	261 AA<br>786 bp	100%	97.96%	CP044098.1	*Citrobacter* Sp.
35	27361	27978	Putative prophage protein	205 AA<br>618 bp	100%	94.01%	CP070177.1	*Klebsiella pneumoniae*
36	27985	28137	Hypothetical protein	50 AA<br>153 bp	100%<br>86%	92.86 %<br>95.38%	NC_049459.1<br>AP022419.1	*Salmonella* phage SW9<br>*Klebsiella pneumoniae*
37	28292	29329	Phage portal protein	345 AA<br>1038 bp	100%	95.57%	LS483465.1	*Salmonella enterica, Serovar* Senftenberg

Supplementary table 5; BIS20 ORF Protein homology with closest phages and prophages in NCBI (BLAST p)

BIS20 ORFs	Putative Function MZ520833	*Escherichia* phage 186 NC_001317.1	*Salmonella* phage SEN1 KT630644.2	*Salmonella* phage SW9 NC_049459.1	*Enterobacteria* phage psp3 AY135486.1	*SEStP* LS483465.1 (675000-706000)	*Citrobacter* Sp. prophage CP056653.1 (1294999-1327000)
2	Terminase ATPase subunit	100%*<br>98.47% ǂ	100%<br>97.63%	99%<br>97.62%	100%<br>97.80%	99%<br>99.32%	100%<br>99.66%
3	Phage Capsid Scaffolding Protein	100%<br>95.09%	100%<br>92.98%	100%<br>92.63%	100%<br>91.93%	99%<br>93.31%	100%<br>96.49%
4	Phage major Capsid Protein P2 family	99%<br>92.94%	100%<br>99.44%	100%<br>99.72%	100%<br>94.94	99%<br>99.4%	100%<br>96.91%
5	Terminase endonuclease subunit	100%<br>98%	100%<br>96.40%	100%<br>95.20%	100%<br>94.40%	99%<br>98.39%	100%<br>97.60%
6	Phage capsid completion protein	100%<br>99.41%	100%<br>95.68%	100%<br>96.45%	100%<br>96.45%	99%<br>99.40%	100%<br>99.41%
7	Phage tail X protein	100%<br>89.71%	100%<br>89.71%	92%<br>90.48%	100%<br>86.76%	98%<br>95.52%	100% 95.59%
8	Phage holin family 2	100%<br>96.97%	100%<br>94.95%	100%<br>95.96%	100%<br>95.96%	98%<br>98.98%	100% 98.99%
9	Lysozyme	100<br>96.39	100<br>97.59	100%<br>97.59%	100<br>96.99	99%<br>95.76%	100%<br>97.59
10	Phage lysis regulatory protein; Spannin	100%<br>94.93%	100<br>92.37	100<br>93.22	100%<br>90.68%	99%<br>97.81%	100%<br>99.28
11	Phage tail completion protein	100%<br>93.59%	100%<br>98.08%	100%<br>98.08%	100%<br>98.08%	100%,<br>98%	99%<br>100%
12	phage virion morphogenesis protein_13	100<br>96	100%<br>92%	100<br>94.67%	100%<br>90.67%	100%<br>97.33%	100<br>96.97%

13	Phage base plate assembly protein	100%<br>91.59%	100%<br>95.79%	100<br>95.33%	100%<br>96.26	100%<br>97.20%	99%<br>96.24%
14	Gene 25 like lysozyme	100%<br>96.55%	100%<br>93.97%	100%<br>95.69%	100%<br>93.10%	99%,<br>96.52%	100%<br>100%
15	Baseplate assembly protein	100%<br>94.39%	100%<br>91.09%	100%<br>92.74%	100%<br>91.09%	99%,<br>97.68%	99%<br>87.75%
16	Prophage function phage tail protein I	98%<br>93.71%	98%<br>84.57%	98%<br>85.14%	98%<br>84%	99%,<br>97.74%	98%<br>66.29%
**17**	**Tail fiber domain containing protein**	**54%<br>73.60%**	**47%<br>75.27%**	**82%<br>75.69%**	**52%<br>66.14%**	**99%,<br>94.68%**	**44%<br>81.71%**
**18**	**Tail fiber assembly protein**	**86%<br>28%**	**37%<br>30.77%**	**84%<br>34.42%**	**84%<br>34.64%**	**100%,<br>96%**	**98%<br>43%**
19	Tail sheath protein	100%<br>95%	100%<br>92.42%	100%<br>94.15%	100%<br>92.93%	99%,<br>99.23%	100%<br>98.47
20	Phage major tail tube protein	100%<br>98.85%	37%<br>98.48%	98%<br>97.67%	98%<br>98.26%	100%,<br>97.20%	99%<br>98.84
21	Hypothetical protein (Putative tail assembly chaperon)	100%<br>99.11%	100%<br>95.54%	100%<br>95.54%	100%<br>96.43%	100%,<br>100%	99%<br>100%
22	Phage tail tape measure protein	100%<br>96%	99%<br>89.67%	99%<br>89.79%	99%<br>89.79%	99%,<br>96.56%	99%<br>96.19%
23	Bacteriophage P2 GPU, tail assembly protein	100%<br>93.83%	100%<br>92.59%	100%<br>92.59%	100%<br>93.21%	100%<br>98.21%	100% 97.53%
24	Phage late control gene D protein	100%<br>96.15%	97%<br>95.26%	98%<br>95.06%	97%<br>96%	99%<br>99.49%	99%<br>99.49%
**25**	**Tyrosine recombinase, Xer C, integrase**	**94%<br>96.67%**	**97%<br>53.43%**	**97%<br>51.14%**	**97%<br>53.43%**	**100%,<br>87.94%**	**97%<br>51.14%**
**26**	**Phage CI-C repressor, lytic- lysogenic switch**	**100%<br>62.44%**	**91%<br>30.43%**	**93%<br>29%**	**90%<br>30.77%**	**100%<br>60.51%**	**94%<br>31%**
**27**	**Hypothetical Protein Putative Excisionase**	**98%<br>70.11%**	**-**	**-**	**-**	**-, -**	**-**

28	Hypothetical Protein CII bacteriophage 186, putative DNA binding protein	100<br>85.88	100%<br>83.53%	100%<br>90.59%	100<br>90.59	100%<br>85.29%	100%<br>91.18%
29	Protein Fil	100%,<br>96.05%	100%,<br>92.11%	100%,<br>90.79%	91.3%, 82%	100<br>96.05%	65%<br>84%
30	DUF 2732 unknown function protein	100%,<br>93.59%	78,<br>81.97	78%,<br>80.33	100,<br>79.49	69%<br>83.33%	78%<br>80.33%
31	DSKa like Zinc finger like domain protein, phage conjugal plasmid C4 (host transcription inhibition)	97%<br>73.61%	98%<br>84.93%	98%<br>84.93%	97%<br>83.33%	98%<br>86.30%	97%<br>93%
32	Replication endonuclease	91%<br>95.54%	98%<br>93.84%	98%<br>95.21%	95%<br>92.76%	99%<br>87.40%	98%<br>88.28%
33	DNA damage inducible protein	93%<br>96.38%	-	-	-	100%,<br>92.52%	--
**34**	**Hypothetical Protein**	**93%<br>91.39%**	**-**	**-**	**-**	**93%<br>92.62%**	**92%<br>87.65%**
**35**	**Putative prophage protein**	**-**	**-**	**-**	**-**	**50%,<br>30.70%**	**-**
**36**	**Hypothetical protein**	**-**	**-**	**-**		**-, -**	**84%, 76.74%**
37	Phage portal protein PBSX family	100%<br>97.11%	95%<br>98.48%	100%<br>96.82%	100%<br>97.11%	99%<br>98.55%	100%<br>96.55%

* Querry coverage percentage in BLAST p

ǂ Similarity index of proteins with BIS20 ORFs amino acid sequence.

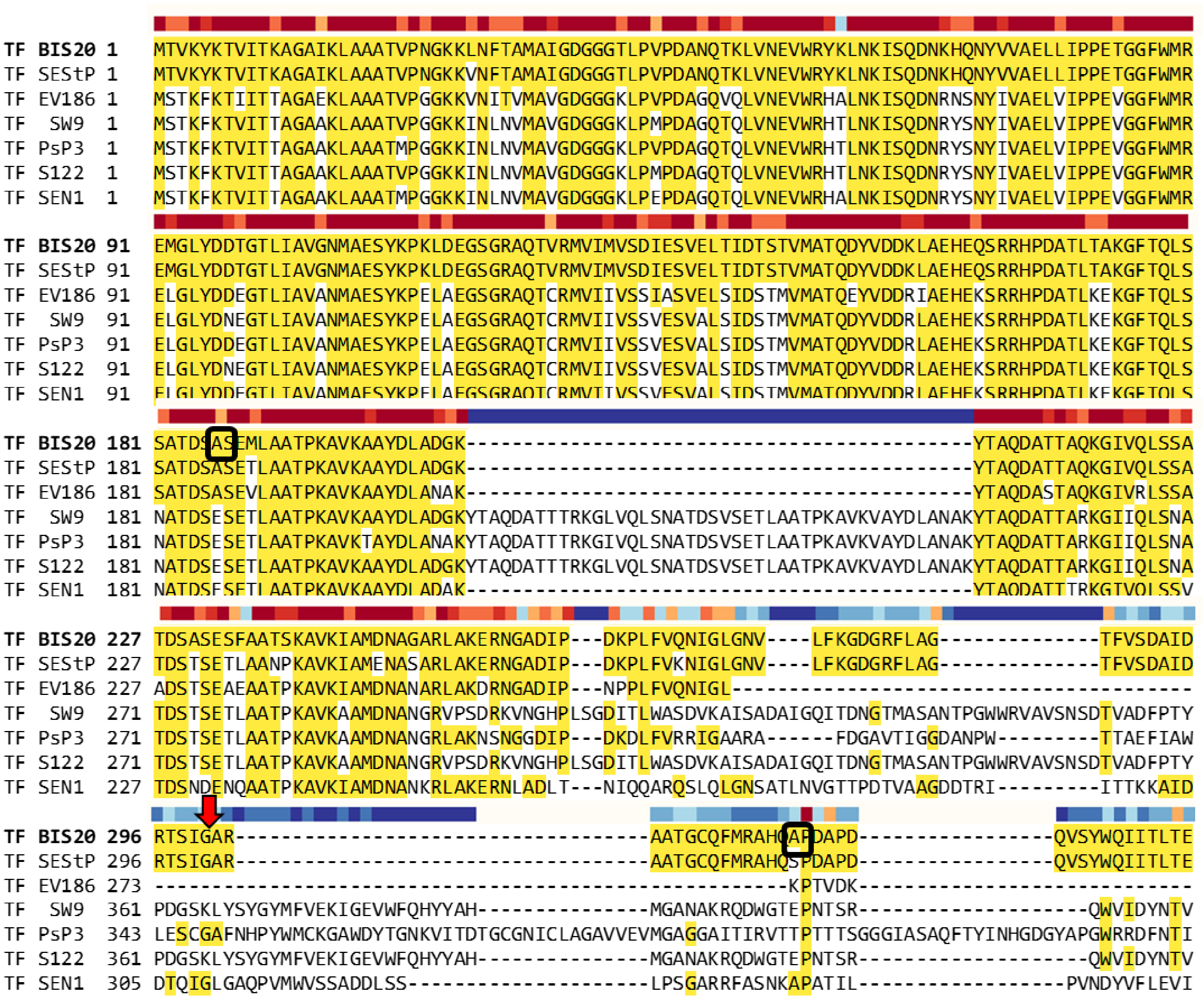
TF BIS20 1 MTVKYKTVITKAGAIKLAAATVPNGKKLNFTAMAIGDGGGTLPVPDANQTKLVNEVWRYKLNKISQDNKHQNYVVAELLIPPETGGFWMR
TF SEStP 1 MTVKYKTVITKAGAIKLAAATVPNGKKVNFTAMAIGDGGGTLPVPDANQTKLVNEVWRYKLNKISQDNKHQNYVVAELLIPPETGGFWMR
TF EV186 1 MSTKFKTIITTAGAEKLAAATVPGGKKVNITVMAVGDGGGKLPVPDAGQVQLVNEVWRHALNKISQDNRNSNYIVAELVIPPEVGGFWMR
TF SW9 1 MSTKFKTVITTAGAAKLAAATVPGGKKINLNVMAVGDGGGKLPMPDAGQTQLVNEVWRHTLNKISQDNRYSNYIVAELVIPPEVGGFWMR
TF PsP3 1 MSTKFKTVITTAGAAKLAAATMPGGKKINLNVMAVGDGGGKLPVPDAGQTQLVNEVWRHTLNKISQDNRYSNYIVAELVIPPEVGGFWMR
TF S122 1 MSTKFKTVITTAGAAKLAAATVPGGKKINLNVMAVGDGGGKLPMPDAGQTQLVNEVWRHTLNKISQDNRYSNYIVAELVIPPEVGGFWMR
TF SEN1 1 MSTKFKTVITTAGAAKLAAATMPGGKKINLNVMAVGDGGGKLPEPDAGQTQLVNEVWRHALNKISQDNRYSNYIVAELVIPPEVGGFWMR
TF BIS20 91 EMGLYDDTGTLIAVGNMAESYKPKLDEGSGRAQTVRMVIMVSDIESVELTIDTSTVMATQDYVDDKLAEHEQSRRHPDATLTAKGFTQLS
TF SEStP 91 EMGLYDDTGTLIAVGNMAESYKPKLDEGSGRAQTVRMVIMVSDIESVELTIDTSTVMATQDYVDDKLAEHEQSRRHPDATLTAKGFTQLS
TF EV186 91 ELGLYDDEGTLIAVANMAESYKPELAEGSGRAQTCRMVIIVSSIASVELSIDSTMVMATQEYVDDRIAEHEKSRRHPDATLKEKGFTQLS
TF SW9 91 ELGLYDNEGTLIAVANMAESYKPELAEGSGRAQTCRMVIIVSSVESVALSIDSTMVMATQDYVDDRLAEHEKSRRHPDATLKEKGFTQLS
TF PsP3 91 ELGLYDDEGTLIAVANMAESYKPELAEGSGRAQTCRMVIIVSSVESVALSIDSTMVMATQDYVDDRLAEHEKSRRHPDATLKEKGFTQLS
TF S122 91 ELGLYDNEGTLIAVANMAESYKPELAEGSGRAQTCRMVIIVSSVESVALSIDSTMVMATQDYVDDRLAEHEKSRRHPDATLKEKGFTQLS
TF SEN1 91 ELGLYDDEGTLIAVANMAESYKPELAEGSGRAQTCRMVIIVSSVESVALSIDSTMVMATQDYVDDRLAEHEKSRRHPDATLKEKGFTQLS
TF BIS20 181 SATDSASEMLAATPKAVKAAYDLADGK--YTAQDATTAQKGIVQLSSA
TF SEStP 181 SATDSASETLAATPKAVKAAYDLADGK--YTAQDATTAQKGIVQLSSA
TF EV186 181 SATDSASEVLAATPKAVKAAYDLANAK--YTAQDASTAQKGIVRLSSA
TF SW9 181 NATDSESETLAATPKAVKAAYDLADGKYTAQDATTTRKGLVQLSNATDSVSETLAATPKAVKVAYDLANAKYTAQDATTARKGIIQLSNA
TF PsP3 181 NATDSESETLAATPKAVKTAYDLANAKYTAQDATTTRKGIVQLSNATDSVSETLAATPKAVKVAYDLANAKYTAQDATTARKGIIQLSNA
TF S122 181 NATDSESETLAATPKAVKAAYDLADGKYTAQDATTTRKGLVQLSNATDSVSETLAATPKAVKVAYDLANAKYTAQDATTARKGIIQLSNA
TF SEN1 181 NATDSESETLAATPKAVKAAYDLADAK--YTAQDATTTRKGIVQLSSV
TF BIS20 227 TDSASESFAATSKAVKIAMDNAGARLAKERNGADIP---DKPLFVQNIGLGNV----LFKGDGRFLAG--------------TFVSDAID
TF SEStP 227 TDSTSETLAANPKAVKIAMENASARLAKERNGADIP---DKPLFVKNIGLGNV----LFKGDGRFLAG--------------TFVSDAID
TF EV186 227 ADSTSEAEAATPKAVKIAMDNANARLAKDRNGADIP---NPPLFVQNIGL--
TF SW9 271 TDSTSETLAATPKAVKAAMDNANGRVPSDRKVNGHPLSGDITLWASDVKAISADAIGQITDNGTMASANTPGWWRVAVSNSDTVADFPTY
TF PsP3 271 TDSTSETLAATPKAVKAAMDNANGRLAKNSNGGDIP---DKDLFVRRIGAARA------FDGAVTIGGDANPW---------TTAEFIAW
TF S122 271 TDSTSETLAATPKAVKAAMDNANGRVPSDRKVNGHPLSGDITLWASDVKAISADAIGQITDNGTMASANTPGWWRVAVSNSDTVADFPTY
TF SEN1 227 TDSNDENQAATPKAVKIAMDNANKRLAKERNLADLT---NIQQARQSLQLGNSATLNVGTTPDTVAAGDDTRI---------ITTKKAID
TF BIS20 296 RTSIGAR------------------------------------AATGCQFMRAHQAPDAPD-----------------QVSYWQIITLTE
TF SEStP 296 RTSIGAR------------------------------------AATGCQFMRAHQSPDAPD-----------------QVSYWQIITLTE
TF EV186 273 ---KPTVDK-----------------------------
TF SW9 361 PDGSKLYSYGYMFVEKIGEVWFQHYYAH---------------MGANAKRQDWGTEPNTSR--------------------QWVIDYNTV
TF PsP3 343 LESCGAFNHPYWMCKGAWDYTGNKVITDTGCGNICLAGAVVEVMGAGGAITIRVTTPTTTSGGGIASAQFTYINHGDGYAPGWRRDFNTI
TF S122 361 PDGSKLYSYGYMFVEKIGEVWFQHYYAH---------------MGANAKRQDWGTEPNTSR--------------------QWVIDYNTV
TF SEN1 305 DTQIGLGAQPVMWVSSADDLSS---------------------LPSGARRFASNKAPATIL------------------PVNDYVFLEVI

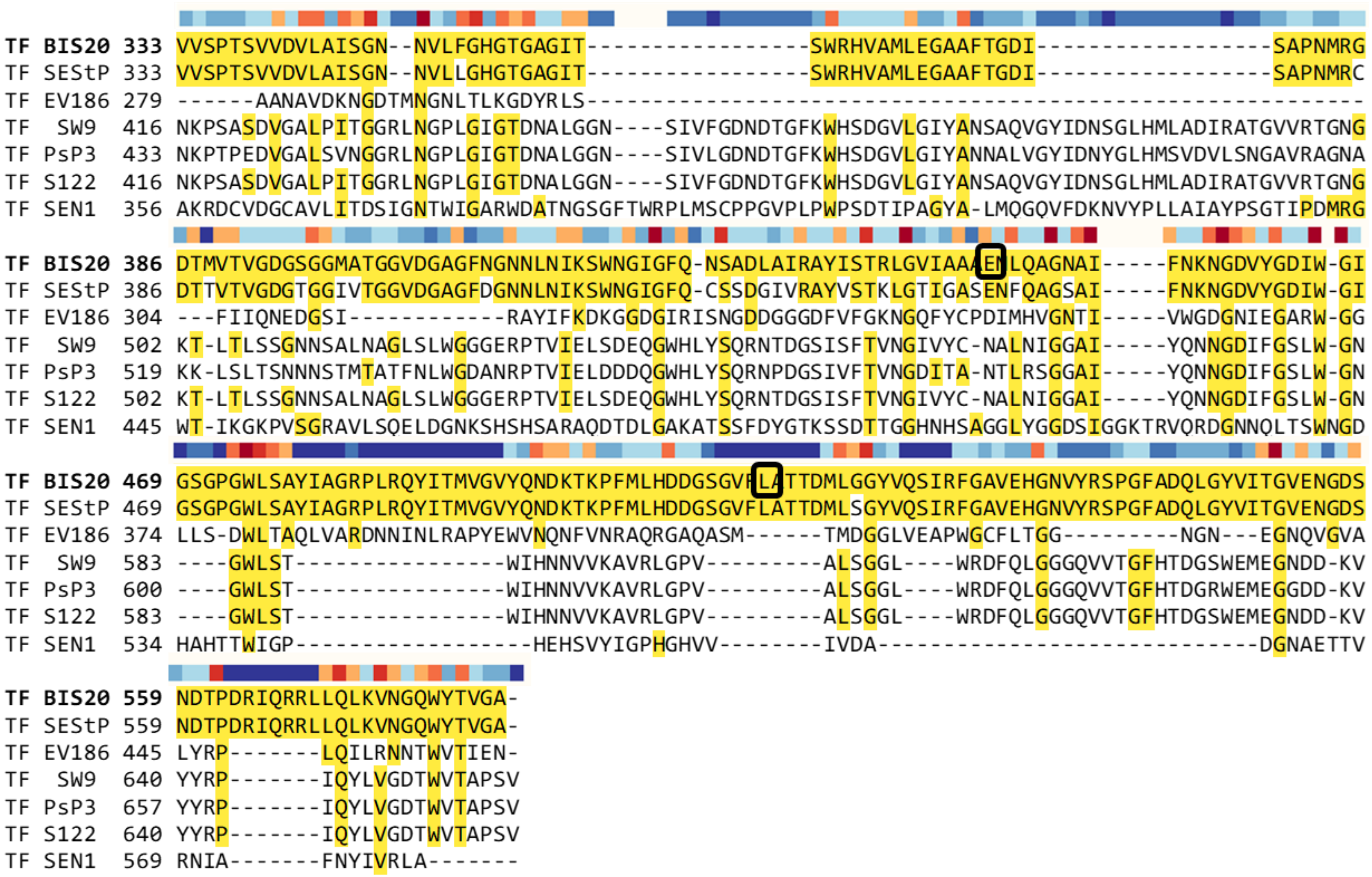

Supplementary Figure 1; Alignment of amino acid sequence of tail fiber gene (ORF17). Tail fiber gene of seven phage and prophage homologs of BIS20 were compared. All sequences were compared with respect to BIS20 sequence. Sequence conservation is represented by colored block on top of amino acids. **Red arrow** represents the best single breakpoint of recombination identified by GARD (http://www.datamonkey.org/GARD/) analysis whereas **black boxes** represent other breakpoints identified by software.

my_phage PhageTerm Analysis

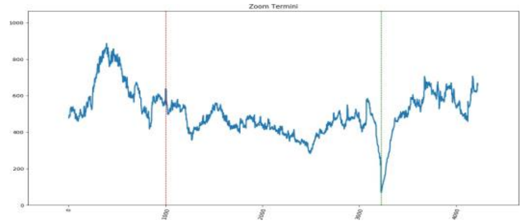

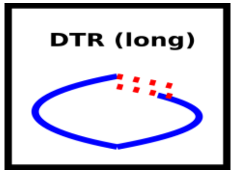

PhageTerm Method

Ends	Left (red)	Right (green)	Permuted	Orientation	Class	Type
Redundant	25888	28114	No	NA	DTR (long)	T5

Direct Terminal Repeats: 2227 bp

Strand	Location	T	pvalue
+	25888	0.28	5.35e-14
	17795	0.24	2.08e-02
	16349	0.24	9.98e-01
	1682	0.22	6.27e-21
	9056	0.22	3.25e-25
-	28114	0.72	4.99e-38
	1690	0.24	1.54e-20
	7911	0.22	1.29e-09
	17803	0.21	1.49e-01
	20799	0.21	1.00e+00

T (Start. Pos. Cov. / Whole Cov.)

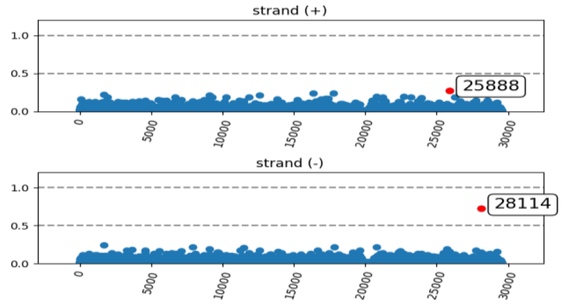

Li's Method

Packaging	Termini	Forward	Reverse	Orientation
OTHER	Preferred	Multiple-Pref. Term.	Multiple-Pref. Term.	Reverse

Strand	Location	SPC	R
+	9056	95	1.0
	21225	82	-
	17795	81	-
	1682	80	-
	5535	64	-
-	28114	140	2.0
	1690	79	-
	9064	69	-
	25896	67	-
	17803	64	-

SPC

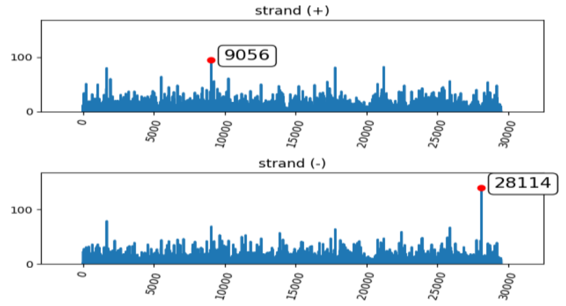

Analysis Methodology

PhageTerm software uses raw reads of a phage sequenced with a sequencing technology using random fragmentation and its genomic reference sequence to determine the termini position. The process starts with the alignment of NGS reads to the phage genome in order to calculate the starting position coverage (SPC), where a hit is given only to the position of the first base in a successfully aligned read (the alignment algorithm uses the lenght of the seed (default: 20) for mapping and does not accept gap or missmatch to speed up the process). Then the program apply 2 distinct scoring methods: i) a statistical approach based on the Gamma law; and ii) a method derived from LI and al. 2014 paper.

General set-up and mapping informations

**Phage Genome**	29512 bp
**Sequencing Reads**	244388
**Mapping Reads**	97 %
OPTIONS	
Mapping Seed	*20*
Surrounding	*20*
Host Analysis	*No*

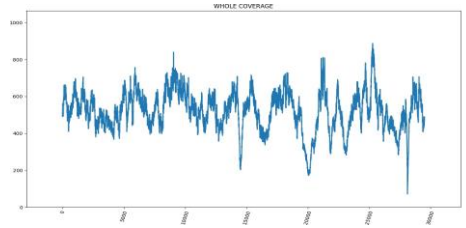

Highest peak of each side coverage graphics

**Whole Coverage Zoom (Left)**	**Whole Coverage Zoom (Right)**

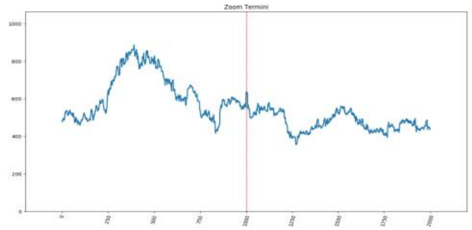

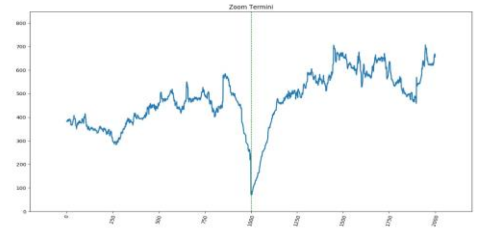

General controls information

**Whole genome coverage**	255	OK
**Weak genome coverage**	0.0 %	OK
**Reads lost during alignment**	2.1 %	OK

i) PhageTerm method

Reads are mapped on the reference to determine the starting position coverage (SPC) as well as the coverage (COV) in each orientation. These values are then used to compute the variable T = SPC / COV. The average value of T at positions along the genome that are not termini is expected to be 1/F, where F is the average fragment size. For the termini that depends of the packaging mode. Cos Phages: no reads should start before the terminus and therefore X=1. DTR phages: for N phages present in the sample, there should be N fragments that start at the terminus and N fragments that cover the edge of the repeat on the other side of the genome as a results T is expected to be 0.5. Pac phages: for N phages in the sample, there should be N/C fragments starting at the pac site, where C is the number of phage genome copies per concatemer. In the same sample N fragments should cover the pac site position, T is expected to be (N/C)/(N+N/C) = 1/(1+C). To assess whether the number of reads starting at a given position along the genome can be considered a significant outlier, PhageTerm first segments the genome according to coverage using a regression tree. A gamma distribution is then fitted to SPC for each segment and an adjusted p-value is computed for each position. Finally if several significant peaks are detected within a small sequence window (default: 20bp), their T values are merged.

**Nearby Termini (Forward / Reverse)**	0 / 0	Peaks localized 20 bases around the maximum

ii) Li's method

The second approach is based on the calculation and interpretation of three specific ratios R1, R2 and R3 as suggested in a previous publication from Li et al. 2014. The first ratio, is calculated as follow: the highest starting frequency found on either the forward or reverse strands is divided by the average starting frequency, R1 = (highest frequency/average frequency). Li's et al. have proposed three possible interpretation of the R1 ratio. First, if R1 < 30, the phage genome does not have any termini, and is either circular or completely permuted and terminally redundant. The second interpretation for R1 is when 30 ≤ R1 ≥ 100, suggesting the presence of preferred termini with terminal redundancy and apparition of partially circular permutations. At last if R1 > 100 that is an indication that at least one fixed termini is present with terminase recognizing a specific site. The two other ratios are R2 and R3 and the calculation is done in a similar manner. R2 is calculated using the highest two frequencies (T1-F and T2-F) found on the forward strand and R3 is calculated using the highest two frequencies (T1-R and T2-R) found on the reverse strand. To calculate these two ratios, we divide the highest frequency by the second highest frequency T2. So R2 = (T1-F / T2-F) and R3 = (T1-R / T2-R). These two ratios are used to analyze termini characteristics on each strand taken individually. Li et al. suggested two possible interpretations for R2 and R3 ratios combine to R1. When R1 < 30 and R2 < 3, we either have no obvious termini on the forward strand, or we have multiple preferred termini on the forward strand, if 30 ≤ R1 ≤ 100. If R2 > 3, it is suggested that there is an obvious unique termini on the forward strand. The same reasoning is applicable for the result of R3. Combining the results for ratios found with this approach, it is possible to make the first prediction for the viral packaging mode of the analyzed phage. A unique obvious termini present at both ends (both R2 and R3 > 3) reveals the presence of a COS mode of packaging. The headful mode of packaging PAC is concluded when we have a single obvious termini only on one strand. A whole coverage around 500X is needed for this method to be reliable.

**Nearby Termini (Forward / Reverse)**	0 / 0	Peaks localized 20 bases around the maximum
**R1 - highest freq./average freq.**	77	Presence of preferred termini with terminal redundancy and apparition of partially circular permutations.
**R2 Forw - highest freq./second freq.**	1	Multiple preferred termini on the forward strand.
**R3 Rev - highest freq./second freq.**	2	Multiple preferred termini on the reverse strand.

Please cite: Sci. Rep. DOI 10.1038/s41598-017-07910-5

Garneau, Depardieu, Fortier, Bikard and Monot. PhageTerm: Determining Bacteriophage Termini and Packaging using NGS data.

Report generated : Wed Feb 2 18:53:59 2022

Supplementary Figure 2; Analysis of phage termini as performed by Phage Term software freely available at https://galaxy.pasteur.fr/?tool. The report indicates the type of termini and probable mode of packaging for BIS20.

GeneMark.hmm PROKARYOTIC (Version 3.26)

Date: Mon Jan 31 07:49:07 2022

Sequence file name: BIS20

Model file name: GeneMark_hmm_heuristic.mod

RBS: false

Model information: Heuristic_model_for_genetic_code_11_and_GC_53

FASTA definition line: empty-fasta-def-line

Gene	Strand	Left End	Right End	Gene Length	Class
1	-	<2	637	636	1
2	-	637	2406	1770	1
3	+	2572	3426	855	1
4	+	3503	4570	1068	1
5	+	4598	5323	726	1
6	+	5416	5922	507	1
7	+	5922	6125	204	1
8	+	6129	6425	297	1
9	+	6412	6909	498	1

10	+	6906	7319	414	1
11	+	7418	7894	477	1
12	+	7887	8336	450	1
13	+	8405	9046	642	1
14	+	9043	9390	348	1
15	+	9397	10305	909	1
16	+	10298	10831	534	1
17	+	10839	12590	1752	1
18	+	12593	13117	525	1
19	+	13248	14426	1179	1
20	+	14442	14963	522	1
21	+	15027	15362	336	1
22	+	15507	17948	2442	1
23	+	17962	18447	486	1
24	+	18444	19613	1170	1
25	-	20167	21210	1044	1
26	-	21210	21797	588	1

27	+	22195	22704	510	1
28	+	22926	23126	201	1
29	+	23193	23426	234	1
30	+	23426	23647	222	1
31	+	23742	25865	2124	1
32	+	26010	26423	414	1
33	+	26453	27238	786	1
34	+	27361	27978	618	1
35	-	28292	29329	1038	1

Supplementary table 6; Gene annotation performed by GeneMark software. The genes were similar as predicted by GAMOLA (Supp. Table 4 and 5) with few exceptions.
